# Single Cell Visualization of Yeast Gene Expression Shows Correlation of Epigenetic Switching between Multiple Heterochromatic Regions through Multiple Generations

**DOI:** 10.1371/journal.pbio.1001601

**Published:** 2013-07-02

**Authors:** Yasunobu Mano, Tetsuya J. Kobayashi, Jun-ichi Nakayama, Hiroyuki Uchida, Masaya Oki

**Affiliations:** 1 Department of Applied Chemistry and Biotechnology, Graduate School of Engineering, University of Fukui, Fukui, Japan; 2 Research Fellow of the Japan Society for the Promotion of Science, Tokyo, Japan; 3 Institute of Industrial Science, The University of Tokyo, Tokyo, Japan; 4 PRESTO, Japan Science and Technology Agency (JST), Saitama, Japan; 5 Laboratory for Chromatin Dynamics, Center for Developmental Biology, RIKEN, Kobe, Japan; 6 Graduate School of Natural Sciences, Nagoya City University, Nagoya, Japan; 7 Research and Education Program for Life Science, University of Fukui, Fukui, Japan; Stowers Institute, United States of America

## Abstract

Differences in gene expression between individual cells can be mediated by epigenetic regulation; thus, methods that enable detailed analyses of single cells are crucial to understanding this phenomenon. In this study, genomic silencing regions of *Saccharomyces cerevisiae* that are subject to epigenetic regulation, including the *HMR*, *HML*, and telomere regions, were investigated using a newly developed single cell analysis method. This method uses fluorescently labeled proteins to track changes in gene expression over multiple generations of a single cell. Epigenetic control of gene expression differed depending on the specific silencing region at which the reporter gene was inserted. Correlations between gene expression at the *HMR*-left and *HMR*-right regions, as well as the *HMR*-right and *HML*-right regions, were observed in the single-cell level; however, no such correlations involving the telomere region were observed. Deletion of the histone acetyltransferase *GCN5* gene from a yeast strain carrying a fluorescent reporter gene at the *HMR*-left region reduced the frequency of changes in gene expression over a generation. The results presented here suggest that epigenetic control within an individual cell is reversible and can be achieved via regulation of histone acetyltransferase activity.

## Abstract 2

A single-cell method allows the assessment of relationships between the dynamic epigenetic behavior of yeast heterochromatin boundaries over multiple generations.

## Author Summary

Although eukaryotic gene repression usually acts on individual genes, cells can also repress larger chromosomal regions via the establishment of a high order chromatin structure called heterochromatin. Once initiated, heterochromatin spreads until halted by a boundary, and in this study we focus on how this boundary is formed. Because the mechanism is epigenetic and can differ from cell to cell, we wanted to assess the dynamics of the process by tracking individual cells over multiple generations. Here we develop a novel method employing protein fluorescence to monitor gene expression at the boundaries of several yeast heterochromatic regions simultaneously. This allows us to assess whether different boundaries within a single cell fluctuate in concert or independently of each other. In addition, we use histone modification mutants to probe the specific types of epigenetic regulation responsible for fluctuations in heterochromatin boundary positioning. Using this method, we show that epigenetic gene expression within individual cells is reversible and that this process is regulated by histone acetylation state. Future work will identify connections between variation in boundary positioning and novel transcription control systems.

## Introduction

The silencing domain in *Saccharomyces cerevisiae* comprises the homothallic mating-type loci *HMR* and *HML*, telomeres, and the rDNA locus. Repression of gene expression in *HMR*, *HML*, and telomere regions is achieved via binding of a protein complex that includes Sir2p, Sir3p, and Sir4p, whereas repression of gene expression of the rDNA region is achieved by binding of Sir2p [1],[2]. Silencing at these regions is halted by boundaries that prevent extension along the entire length of the chromosome. Three models of boundary formation have recently been proposed. In these models, boundary formation depends on the DNA sequence [3], is controlled by histone modification [4], or depends on the interaction between nuclear pores and chromosomes [5]. Most of the boundaries flanking the regions within the yeast silencing domain have been determined; *tRNA* is located to the right of the *HMR* [6]–[8], while the *CHA1* promoter is located to the right of the *HML* [9] and LB (Left Boundary) to the left of the *HML* [10], the telomere is flanked by STARs (subtelomeric antisilencing regions) [11], and the rDNA region is flanked by *tRNA* and *Ty-LTR* [12]. However, the structure of the boundary positioned on the left of *HMR* has not yet been elucidated.

A previous study demonstrated that insertion of a reporter gene into the telomere boundary region of yeast produces the position effect variegation (PEV) phenotype [13], indicating that the silencing region within the telomere boundary in an individual cell can expand or shrink and that gene expression in this region is regulated by epigenetic control. When the *ADE2* gene is used as a reporter to analyze the PEV phenotype, yeast cells bearing a telomere-linked gene produce colonies with both red and white sectors [13]. In previous studies that used the *URA3* gene as a reporter to analyze the PEV phenotype, the gene was inserted close to the telomere, *HMR*, *HML*, or rDNA region, which are known to comprise the silencing region in *S. cerevisiae* [6],[10],[14]. The degree of repression of the *URA3* gene inserted at different sites within the region located to the right of the *HML* (*HML*-right) is related to the distance between the inserted promoter and the cis-acting I-silencer sequence that flanks the *HML* region; the PEV phenotype is generated when the *URA3* gene is inserted close to the right side of the I-silencer sequence [15]. These data suggest that the state of gene expression can be epigenetically altered in individual cells; however, the studies described above were restricted to examining yeast colonies and therefore could not measure gene expression in individual cells.

One way to perform single cell analysis with *S. cerevisiae* is to conduct a pedigree assay. This technique was previously used to show that Sir1p is involved in the epigenetic control of gene expression [16] and that the deletion of the *dpb3* or *dpb4* genes, which encode components of DNA polymerase ε, alters the epigenetic switching rate (the rate of change from the active state to the silent state) in individual yeast cells [17]. The histone modification enzymes Dot1p and Set1p, and chromatin assembly factor I, also alter the epigenetic switching rate [18],[19]. Recently, a new approach to single cell analysis of yeast, which uses a fluorescent protein to analyze changes in epigenetic gene expression, was reported. This technique was used to show that the *HMR* and *HML* loci behave independently within a single cell, demonstrating that heterochromatin formation is locus autonomous [20]. However, previous studies of single yeast cells using this method were performed over only a few generations.

This study describes the development of a new method of single cell analysis that employs protein fluorescence to detect changes in the epigenetic control of gene expression for more than 10 generations of protein in yeast cells. The analysis method was used to demonstrate that epigenetic gene expression within an individual yeast cell is reversible and is regulated by histone acetyltransferase.

## Results

### The Spread of the Silencing Effect Differs Between the Left and Right Sides of the *HMR*, the Right Side of the *HML*, and the Telomere

**Figure 1:**
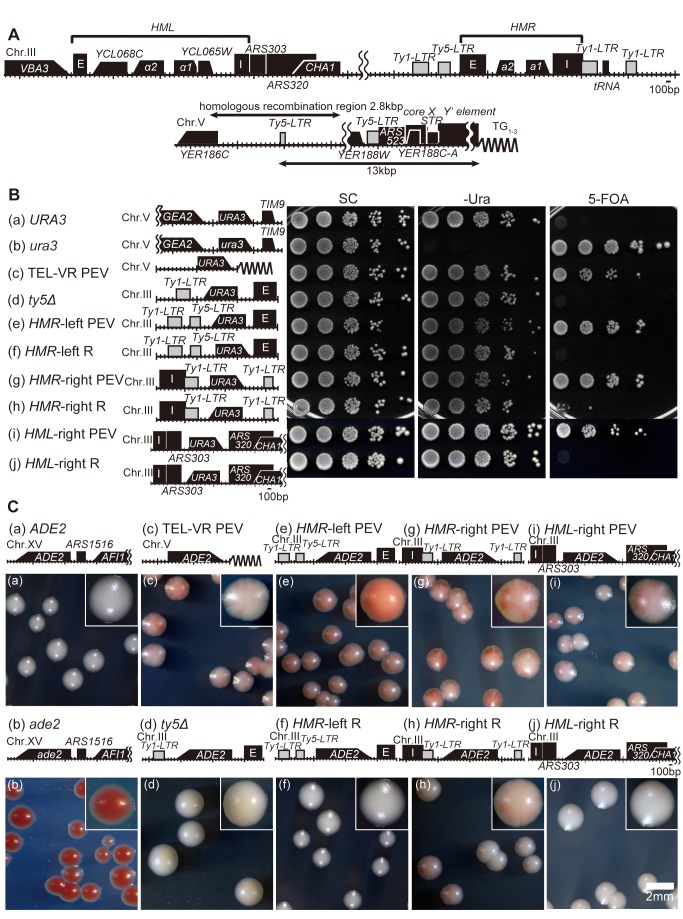
Position effects of the *HMR*, *HML*, and telomere regions. (A) Configuration of the *HMR*, *HML*, and telomere regions. (B) Viability of *S. cerevisiae* strains in which the *URA3* gene was inserted at the indicated regions. The *URA3* (FUY18) (a) and *ura3* (FUY31) (b) strains were used as positive and negative controls. In (c), the *URA3* gene was inserted at the telomere region (TEL-VR PEV:FUY323); in (d), (e), and (f), the insertion was at the *HMR*-left region (*ty5Δ*:FUY324, *HMR*-left PEV:FUY316, *HMR*-left R:FUY325); in (g) and (h), the insertion was at the *HMR*-right region (*HMR*-right PEV:FUY326, *HMR*-right R:FUY327); and in (i) and (j), the insertion was at the *HML*-right region (*HML*-right PEV:FUY782, *HML*-right R:FUY783). Each strain was grown to a final density of A_600_ = 1.0, and then 10-fold serial dilutions were spotted onto complete synthetic medium (SC), synthetic medium lacking uracil (–Ura), or synthetic medium containing 5-FOA. Plates were incubated for 2–3 d at 30°C. (C) A colony color assay of yeast cells carrying the telomere-linked *ADE2* gene. *ADE2* (FUY31) (a) and *ade2* (FUY32) (b) strains were used as positive and negative controls, respectively. In (c), the *ADE2* gene was inserted at the telomere region (TEL-VR PEV, FUY328); in (d), (e), and (f), the insertion was at the *HMR*-left region (*ty5Δ*, FUY152; *HMR*-left PEV, FUY329; *HMR*-left R, FUY330); in (g) and (h), the insertion was at the *HMR*-right region (*HMR*-right PEV, FUY331; *HMR*-right R, FUY332); and in (i) and (j), the insert was at the *HML*-right region (*HML*-right PEV, FUY784; *HML*-right R, FUY785). Freshly grown yeast cells were spread onto SC plates (containing 10 µg/ml adenine) and then incubated at 30°C for a further 2–3 d. Scale bar, 2 mm.

The *URA3* and *ADE2* genes were used as reporters to determine whether silencing from the *HMR*, *HML*, and telomere regions in *S. cerevisiae* occurs in a coordinated manner (Figure 1A). A yeast strain expressing the *URA3* gene grew on medium lacking uracil but was unable to grow on medium containing 5-fluoroorotic acid (5-FOA). By contrast, when *URA3* expression was repressed, yeast could not grow on medium lacking uracil but were able to grow on 5-FOA medium (Figure 1B (a,b)), as reported previously [21]. Yeast cells in which the *URA3* gene was inserted close to the telomere displayed a PEV phenotype, as indicated by growth on both types of medium (Figure 1B (c)), as reported previously [13]. White or red colonies were formed when the *ADE2* gene was expressed or repressed, respectively (Figure 1C (a,b)). Insertion of the *ADE2* gene close to the telomere produced a PEV phenotype, as indicated by the growth of yeast colonies with both red and white sectors (Figure 1C (c)), as reported previously [13].

The *ty5Δ* strain, in which the *Ty5-LTR* in the *HMR*-left boundary region was replaced with the *URA3* gene, was then constructed and a spot assay was performed. The *ty5Δ* yeast grew on medium lacking uracil but barely grew on 5-FOA plates (Figure 1B (d)). The *HMR*-left PEV strain, in which the *URA3* gene was inserted closer to the E-silencer than it was in the *ty5Δ* strain, and the *HMR*-left R strain, which contained the *URA3* promoter positioned in the opposite direction to that in the *HMR*-left PEV strain, were then constructed. The *HMR*-left PEV strain displayed the same PEV phenotype as yeast containing the *URA3* gene close to the telomere (Figure 1B (e)); however, the *HMR*-left R strain did not show this phenotype (Figure 1B (f)). The *HMR*-right PEV and *HMR*-right R strains were constructed in the same manner as the *HMR*-left strains; in both of these constructs, *tRNA* in the *HMR*-right boundary region was replaced with the *URA3* gene. The *HMR*-right PEV strain, in which the *URA3* gene was inserted the same distance from the I-silencer as in the *HMR*-right R strain, displayed the PEV phenotype (Figure 1B (g,h)); however, the *HMR*-right R strain did not. The *HML*-right PEV and *HML*-right R strains were then constructed by inserting the *URA*3 gene downstream of the I-silencer in the *HML*-right boundary region. As expected, the *HML*-right PEV strain displayed the PEV phenotype (Figure 1B (i,j)), but the *HML*-right R strain did not. These data agree with those reported previously [15].

A set of similar experiments that utilized the *ADE2* gene as a reporter instead of *URA3* was then performed (Figure 1C). In these experiments, the *ty5Δ* strain produced a white colony (Figure 1C (d)), and the *HMR*-left PEV strain produced a light pink colony (Figure 1C (e)), suggesting an increased frequency of epigenetic switching in this strain. The *HMR*-left R strain produced a white colony (Figure 1C (f)). The *HMR*-right PEV strain produced a pink colony with a red and white sector, suggesting that these yeast cells retained the same expression state over several generations (Figure 1C (g)), while the *HMR*-right R strain produced a white colony with an inside slightly sectored (Figure 1C (h)). The *HML*-right PEV strain produced a pink colony with an inside sectored (Figure 1C (i)) and the *HML*-right R strain produced a white colony (Figure 1C (j)).

Taken together, these data suggest that the spread of the silencing region in *S. cerevisiae* differs depending on whether the inserted gene is positioned at the *HMR*-left, *HMR*-right, *HML*-right, or telomere region. The data also suggest that changes in epigenetic expression are regulated in individual cells, which highlights the importance of tracking changes in gene expression within a single cell rather than a mixed population of cells.

### Changes in Epigenetic Gene Expression Are Regulated in a Locus-Specific Manner

**Figure 2:**
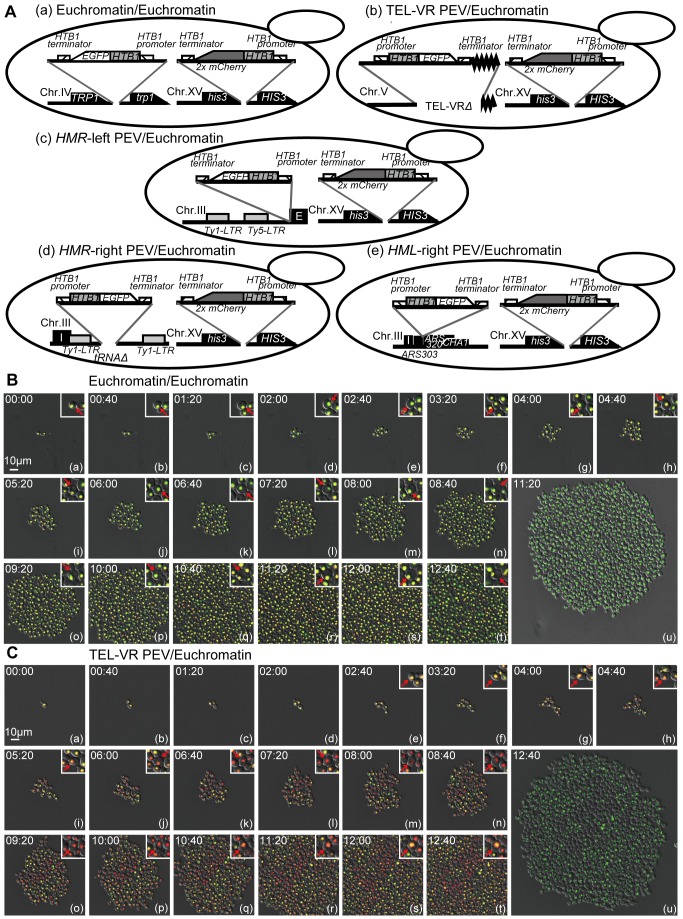
Gene expression states in PEV strains assessed by time-lapse imaging of single yeast cells. (A) Schematic illustration of the yeast strains expressing EGFP and mCherry. Time-lapse images of the Euchromatin/Euchromatin (FUY257) (B) and TEL-VR PEV/Euchromatin (FUY355) (C) strains expressing *HTB1-EGFP* (green fluorescence) and *HTB1-2x mCherry* (red fluorescence). Overlap of the two fluorescent markers is indicated by yellow coloring. Image stacks were taken at 40 min intervals over a 12 h period and the differential interference contrast (DIC) image was merged. The larger panel shows a wide focus image in which only the *HTB1-EGFP* signal is displayed. Single cells are shown in the insets at the top right corner of each panel; the red arrowhead indicates the same cell in each image. Scale bar, 10 µm. See also Movies S1 and S2.

**Figure 3:**
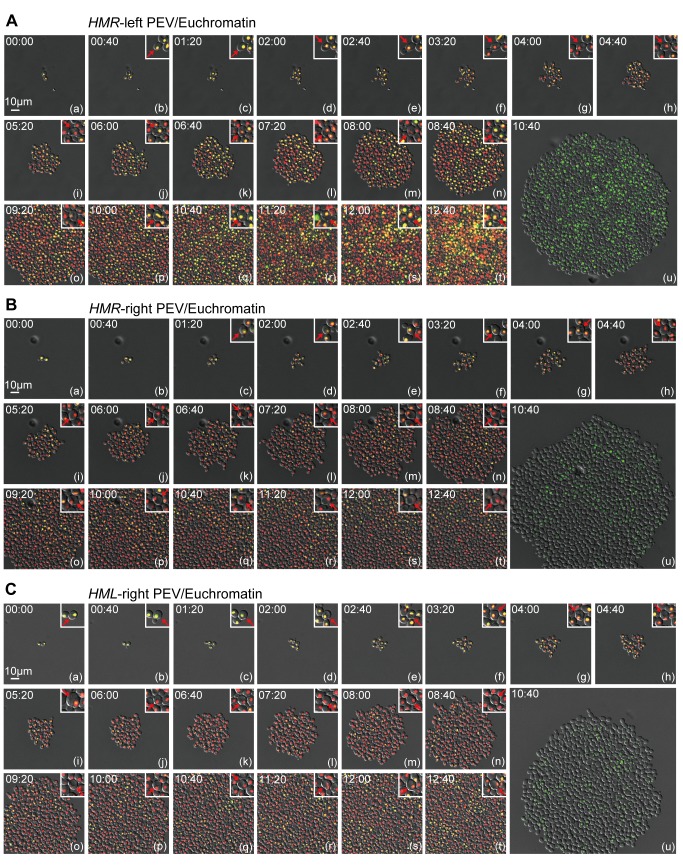
Gene expression states in PEV strains assessed by time-lapse imaging of single yeast cells. Time-lapse images of the *HMR*-left PEV/Euchromatin (FUY263) (A), *HMR*-right PEV/Euchromatin (FUY356) (B), and *HML*-right PEV/Euchromatin (FUY795) (C) strains expressing *HTB1-EGFP* (green fluorescence) and *HTB1-2x mCherry* (red fluorescence), same as Figure 2. See also Movies S3.

To examine gene expression changes in single cells, we developed a new analysis system that utilizes expression of fluorescent proteins. Using this method, a single cell is placed in the center of the field of vision of a microscope and changes in epigenetic gene expression that occur during cell division are followed using time-lapse imaging. Five new yeast strains were constructed to precisely measure the fluorescence intensity in a single cell (Figure 2A). In all strains, the mCherry-tagged *HTB1* gene (*HTB1-2x mCherry*) was inserted into the euchromatin *HIS3* locus on chromosome XV. The control strain (Euchromatin/Euchromatin, FUY257) contained the *EGFP*-tagged *HTB1* gene (*HTB1-EGFP*) inserted into the euchromatin *TRP1* locus of chromosome IV. The TEL-VR PEV/Euchromatin (FUY355) strain contained *HTB1-EGFP* inserted into the telomere on the right side of chromosome V. The *HMR*-left PEV/Euchromatin (FUY263) and *HMR*-right PEV/Euchromatin (FUY356) strains contained *HTB1-EGFP* inserted into the *HMR*-left region or the *tRNA* of the *HMR*-right region on chromosome III, respectively. The *HML*-right PEV/Euchromatin (FUY795) strain contained *HTB1-EGFP* inserted into the *HML*-right region on chromosome III. The EGFP signal was normalized to the mCherry signal to correct for differences in fluorescence intensity caused by gaps in focus. Time-lapse analysis of the Euchromatin/Euchromatin strain revealed that the EGFP and mCherry fluorescent signals were persistent and always coincided with yeast cell division (Figure 2B, Movies S1 and S2). Time-lapse experiments were then performed using the TEL-VR PEV/Euchromatin (Figure 2C), *HMR*-left PEV/Euchromatin (Figure 3A, Movie S3), *HMR*-right PEV/Euchromatin (Figure 3B), and *HML*-right PEV/Euchromatin (Figure 3C) strains. For these strains, the mCherry fluorescent signal did not disappear upon repeated cell division; however, the EGFP fluorescent signal did disappear, although it returned in some progeny upon continued cell division.

**Figure 4:**
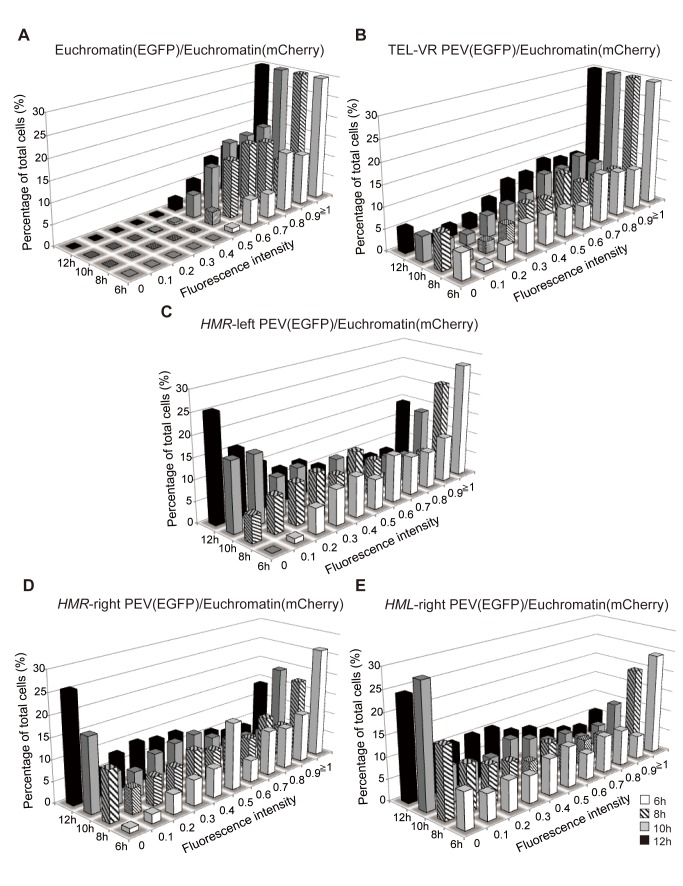
Fluorescence intensity at different time points in the extended time-lapse experiments. (A−E) Histograms showing the fluorescence intensities of the Euchromatin/Euchromatin (FUY257) (A), TEL-VR PEV/Euchromatin (FUY355) (B), *HMR*-left PEV/Euchromatin (FUY263) (C), *HMR*-right PEV/Euchromatin (FUY356) (D), and *HML*-right PEV/Euchromatin (FUY795) (E) cells in the field of vision at 6, 8, 10, and 12 h. Data represent the average of *n* = 3 independent time-lapse experiments. Data were normalized to the highest fluorescence intensity observed at each time-point (maximum fluorescence intensity of 1) and show the ratio of EGFP intensity to mCherry intensity.

Next, the fluorescence intensities of the five strains expressing EGFP and mCherry were measured in all fields of vision at 6, 8, 10, and 12 h after cell division began. Three independent extended time-lapse experiments were performed for each strain and the intensities of the cells within each field of vision were normalized to both the highest level of fluorescence observed at each time-point and the mCherry signal (Figures 4A−E). The fluorescence intensity of the Euchromatin/Euchromatin strain (Figure 4A) and TEL-VR PEV/Euchromatin (Figure 4B) were fairly stable across the time-course, but a gradual decrease in fluorescence intensity was observed for the *HMR*-left PEV/Euchromatin (Figure 4C), *HMR*-right PEV/Euchromatin (Figure 4D), and *HML*-right PEV/Euchromatin (Figure 4E) strains. These data suggest that the spread of gene silencing was altered by repeated cell division and that expression of *HTB1-EGFP* varied within an individual cell. The *HTB1* gene is only active during the S-phase of cell division; therefore, to ensure that the changes in fluorescence observed in the previous experiments were not attributable to properties inherent to the reporter genes, similar experiments were performed using the constitutive *URA3* promoter and *EGFP* as the reporter gene (*NLS-3xEGFP*). Similar to the results observed for the *HTB1-EGFP* gene, these experiments also revealed reversible epigenetic changes in gene expression (Figure S1), suggesting that the changes in fluorescence observed were general phenomena and were independent of the specific reporter gene used. The stability of the expression levels of the two fluorescently labeled reporter genes (*HTB1-EGFP* and *NLS-3xEGFP*) was measured by exposing cells to cycloheximide to inhibit protein synthesis. When the reporter gene was present in either the euchromatin or *HMR*-left PEV region, the EGFP signal was reduced by 50% after 2 h treatment with cycloheximide, which corresponds to the doubling time of yeast (unpublished data). This result is similar to those of other reports [20] and suggests that protein turnover was sufficiently rapid to measure transition in the epigenetic state.

**Figure 5:**
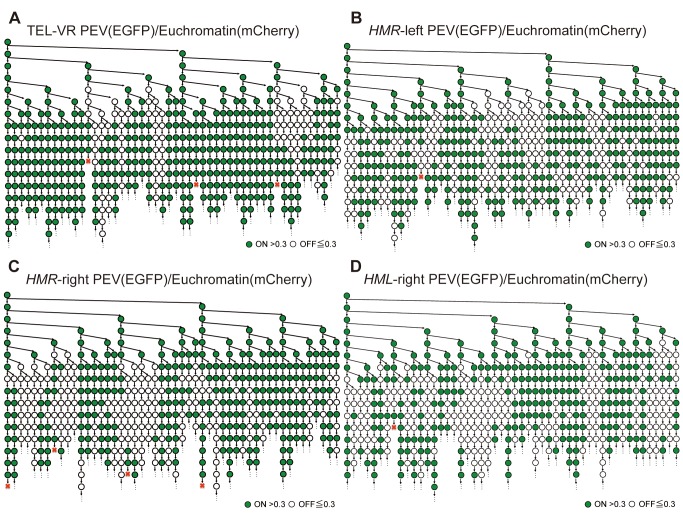
Representative expression lineage trees derived from a single cell. (A−D) The fluorescence intensities of single cells from the TEL-VR PEV/Euchromatin (FUY355) (A), *HMR*-left PEV/Euchromatin (FUY263) (B), *HMR*-right PEV/Euchromatin (FUY356) (C), and *HML*-right PEV/Euchromatin (FUY795) (D) strains were tracked in real time. The highest positioned circle on the left side of each panel indicates the first single cell; vertical arrows indicate the division and lineage of the mothers, whereas downward or horizontal arrows identify the daughter cells of specific mothers. Green cells indicate a fluorescence intensity >0.3 and white cells indicate an intensity <0.3. Dead cells are indicated by a red X. Not every cell is shown on this tree because of spatial constraints. Every cell was counted and the data are summarized in Table S3.

Changes in gene expression were monitored by measuring the fluorescence intensity of single cells from the TEL-VR PEV/Euchromatin (Figure 5A, Table S3A), *HMR*-left PEV/Euchromatin (Figure 5B, Table S3B), *HMR*-right PEV/Euchromatin (Figure 5C, Table S3C), and *HML*-right PEV/Euchromatin (Figure 5D, Table S3D) strains in real time. When *HTB1-EGFP* was inserted close to the telomere, the same gene expression status (either ON or OFF) was maintained for several generations (Figure 5A). However, changes in epigenetic gene expression were less maintained and occurred randomly when *HTB1-EGFP* was inserted on the left side of *HMR* (Figure 5B). The results for the strains containing *HTB1-EGFP* at *HMR*-right or *HML*-right regions were more stable across multiple generations than *HMR*-left PEV/Euchromatin strain (Figure 5C and 5D). These results were similar to those shown in Figure 1, which were obtained using the *ADE2* reporter gene. The phenotype of the cells containing the insert at the *HMR*-left region was a pink colony, suggesting an increased frequency of epigenetic switching in these cells. Conversely, cells in which the insert was positioned close to the telomere with distinct sector and on the right of the *HMR* or *HML* regions produced pink colonies with sectors. These data indicate that the formation of sectors requires maintenance of the same expression status for several generations, whereas pink colonies are produced when the rate of switching of marker gene expression between ON and OFF increases.

Statistical analyses were performed to confirm the results of the single cell measurements. The frequencies of transition events between the ON and OFF gene expression states were calculated and a permutation test was used to determine the reproducibility across independent experiments for the *HMR*-left PEV (Table S4) and TEL-VR PEV (Table S5) strains. Differences in the existence ratio of the ON and OFF states between these independent experiments were observed, but the frequency of change from the ON to OFF state and from the OFF to ON state were reproducible. Therefore, statistical analyses of the data for all four strains (Figure 5) were performed to determine whether a similar or different regularity system governed epigenetic gene expression at an individual region (Tables 1 and 2).

**Table 1:** Statistical test for state-transition at Telomere and *HM* region.

	Frequencies of ON and OFF Cells		Frequencies of ON to ON and ON to OFF Transitions		Frequencies of OFF to ON and OFF to OFF Transitions	
	ON	OFF	ON→ON	ON→OFF	OFF→ON	OFF→OFF
TEL-VR PEV	382	78	326	13	18	53
*HMR*-left PEV	317	143	238	49	42	81
*HMR*-right PEV	299	165	225	46	35	108
*HML*-right PEV	275	189	192	55	42	125

	Ratio of ON and OFF Cells		Ratio of ON to ON and ON to OFF Transitions		Ratio of OFF to ON and OFF to OFF Transitions	
	ON	OFF	ON→ON	ON→OFF	OFF→ON	OFF→OFF
TEL-VR PEV	0.83	0.17	0.962	0.0383	0.254	0.746
*HMR*-left PEV	0.689	0.311	0.829	0.171	0.341	0.659
*HMR*-right PEV	0.644	0.356	0.83	0.17	0.245	0.755
*HML*-right PEV	0.593	0.407	0.777	0.223	0.251	0.749

**Table 2:** *p* Values obtained with state and transition frequencies.

	TEL-VR PEV	*HMR*-Left PEV	*HMR*-Right PEV	*HML*-Right PEV
***p*** ** Value obtained with ON/OFF state frequencies**				
TEL-VR PEV	0.534992496			
*HMR*-left PEV	3.42171E-07	0.528388567		
*HMR*-right PEV	7.73349E-11	0.084927542	0.527332724	
*HML*-right PEV	6.77245E-16	0.001394706	0.060023381	0.526631104
***p*** ** Value obtained with ON to ON and ON to OFF transition frequencies**				
TEL-VR PEV	0.579019299			
*HMR*-left PEV	2.07143E-08	0.544136987		
*HMR*-right PEV	3.70702E-08	0.532772173	0.545518554	
*HML*-right PEV	4.58505E-12	0.080653092	0.079654163	0.543038944
***p*** ** Value obtained with OFF to ON and OFF to OFF transition frequencies**				
TEL-VR PEV	0.576379154			
*HMR*-left PEV	0.132011901	0.553451403		
*HMR*-right PEV	0.507432262	0.055062242	0.554656669	
*HML*-right PEV	0.547467161	0.062313761	0.498653284	0.550150113

Compared with the *HMR*-left, *HMR*-right, and *HML*-right strains, few TEL-VR PEV cells were in the OFF state and the ratio of cells that varied from ON to OFF was also low. However, the changes from the OFF to ON state were not significantly different across these four strains. In addition, although the ratios of ON and OFF cells, as well as the OFF to ON transition frequencies, were slightly different between the *HMR*-left and *HMR*-right strains, these differences were not statistically significant. The change ratio from the ON to OFF state was comparable for these two strains. Furthermore, although the ratio of ON and OFF cells, as well as the change ratio from the ON to OFF and OFF to ON states, differed slightly between the *HMR*-left and *HML*-right strains, these changes were not statistically significant. When comparing the *HMR*-right and *HML*-right strains, the ratio of ON and OFF cells as well as the change ratio from the ON to OFF states were slightly different; however, these changes were also not statistically significant. The change ratio from the OFF to ON state was comparable for these two strains.

In these experiments, we found that the telomere and the *HM* region had very different epigenetic regularity systems, and that *HMR* and *HML* were not perfect much, but they had some similar epigenetic regulation system.

### Changes in Gene Expression Depend on the Sir Protein and the Acetylation Status of Histones

**Figure 6:**
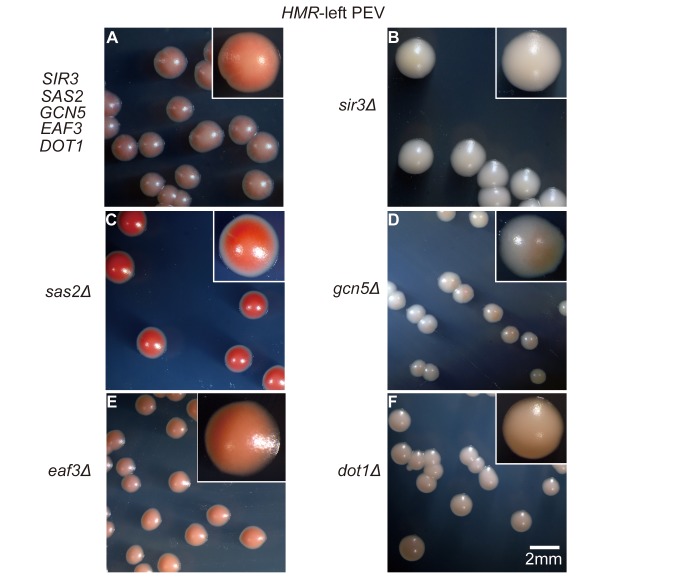
Position effects at the *HMR*-left region assessed using strains containing disrupted histone modification enzyme genes. The *ADE2* gene was inserted into the *HMR*-left region of various deletion strains and a colony color assay was performed. The control strain was *HMR*-left PEV (FUY329) (A) and the deletion strains were *HMR*-left PEV+*sir3Δ* (FUY341) (B), *HMR*-left PEV+*sas2Δ* (FUY346) (C), *HMR*-left PEV+*gcn5Δ* (FUY791) (D), *HMR*-left PEV+*eaf3Δ* (FUY814) (E), and *HMR*-left PEV+*dot1Δ* (FUY820) (F). Freshly grown yeast cells were spread onto SC plates (containing 10 µg/ml adenine) and were incubated at 30°C for 2–3 d. Scale bar, 2 mm.

The Sas2p protein contributes to silencing of the *HMR* region [22], and the spread of the silencing region in the telomere is dependent on the histone modification state. Histone modification is achieved by the histone deacetylase activity of Sir2p and the histone acetyltransferase activity of Sas2p, which acetylates H4 at lysine 16 [23],[24]. In addition, tRNA and histone acetyltransferase are important for the production of a boundary at the *HMR*-right region [7],[25]. Therefore, we analyzed whether the spread of the silencing region at the *HMR* and *HML* depends on the histone modification state, as it does at the telomere. Using *ADE2* as a reporter gene, the following yeast strains in which the *SIR3* gene was disrupted were constructed: *HMR*-left PEV+*sir3Δ*, *HMR*-right PEV+*sir3Δ*, and *HML*-right PEV+*sir3Δ*. The following strains in which the *SAS2* gene was disrupted were also constructed: *HMR*-left PEV+*sas2Δ*, *HMR*-right PEV+*sas2Δ*, and *HML*-right PEV+*sas2Δ*. As controls, TEL-VR PEV strains in which the *SIR3* or *SAS2* gene was disrupted and the *ADE2* gene was inserted at the telomere were also generated. Images of the *HMR*-left PEV strains are shown in Figure 6; images of all other strains are shown in Figure S2. Disruption of the *SIR3* gene in all PEV strains tested produced colonies that were whiter than those produced by the corresponding wild-type PEV strains. By contrast, disruption of the *SAS2* gene produced colonies that were redder than the corresponding wild-type PEV strains. These data suggest that, similar to the telomere, the spread of silencing at the *HMR* and *HML* regions also depends on the histone modification status [4],[23],[24].

### Regulation of Epigenetic Gene Expression Depends on the Activity of a Histone Modification Enzyme

**Figure 7:**
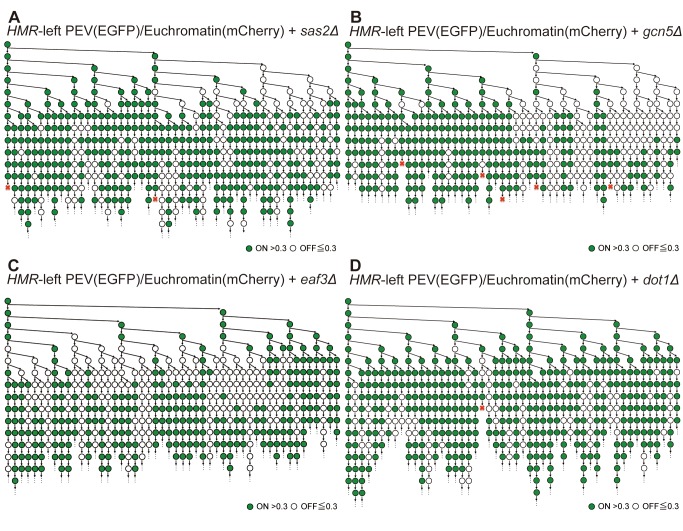
Representative expression lineage trees derived from a single cell of histone modification enzyme deletion strains. (A−D) The fluorescence intensities of single cells from *HMR*-left PEV/Euchromatin+*sas2Δ* (FUY358) (A), *HMR*-left PEV/Euchromatin+*gcn5Δ* (FUY797) (B), *HMR*-left PEV/Euchromatin+*eaf3Δ* (FUY799) (C), and *HMR*-left PEV/Euchromatin+*dot1Δ* (FUY801) (D) strains were tracked in real time. The highest positioned circle on the left side of each panel indicates the first single cell; vertical arrows indicate the division and lineage of the mothers, whereas downward or horizontal arrows identify the daughter cells of specific mothers. Green cells indicate a fluorescence intensity >0.3 and white cells indicate an intensity <0.3. Dead cells are indicated by a red X. Not every cell is shown on this tree because of spatial constraints. Every cell was counted and the data are summarized in Table S6.

To understand why differences in the spread of silencing were observed when the marker gene was inserted to the right or left of the *HMR* locus, and whether this difference is genetically controlled, we focused on a histone modification enzyme that was previously isolated by our group using genome-wide boundary screening [26]. Single cell time-lapse experiments were performed using the *sas2* deletion strains, Euchromatin/Euchromatin+*sas2Δ* and *HMR*-left PEV/Euchromatin+*sas2Δ* (Figure 7A and Table S6A), and then statistical analyses of the data were performed. Small but statistically significant changes in the ratio of ON and OFF cells and the ratio of OFF to ON transitions between the *sas2Δ* and corresponding wild-type strains were observed (5% confidence interval). However, the ratio of ON to OFF transitions was not affected by deletion of the *SAS2* gene (Tables 3 and 4). Although the results were not statistically significant, when we focused on the specific mother cell of the lineage tree, the frequency of the change in the epigenetic gene expression state across generations was increased for some cells (Figure 7A). These data suggested that the *sas2Δ* strain did not undergo a dramatic change in epigenetic regulation, but that *SAS2* might be involved in the regulation of the frequency of change in epigenetic gene expression.

**Table 3:** Statistical test for state-transition with histone modification mutants.

	Frequencies of ON and OFF Cells		Frequencies of ON to ON and ON to OFF Transitions		Frequencies of OFF to ON and OFF to OFF transitions	
	ON	OFF	ON→ON	ON→OFF	OFF→ON	OFF→OFF
*HMR*-left PEV	317	143	238	49	42	81
*HMR*-left PEV sas2Δ	334	119	253	48	47	55
*HMR*-left PEV gcn5Δ	262	129	211	14	24	92
*HMR*-left PEV eaf3Δ	271	168	211	20	36	122
*HMR*-left PEV dot1Δ	373	80	301	34	25	43

	Ratio of ON and OFF Cells		Ratio of ON to ON and ON to OFF Transitions		Ratio of OFF to ON and OFF to OFF Transitions	
	ON	OFF	ON→ON	ON→OFF	OFF→ON	OFF→OFF
HMR-left PEV	0.689	0.311	0.829	0.171	0.341	0.659
*HMR*-left PEV sas2Δ	0.737	0.263	0.841	0.159	0.461	0.539
*HMR*-left PEV gcn5Δ	0.67	0.33	0.938	0.0622	0.207	0.793
*HMR*-left PEV eaf3Δ	0.617	0.383	0.913	0.0866	0.228	0.772
*HMR*-left PEV dot1Δ	0.823	0.177	0.899	0.101	0.368	0.632

**Table 4:** *p* Values obtained with state and transition frequencies.

	*HMR*-Left PEV	*HMR*-Left PEV sas2Δ	*HMR*-Left PEV gcn5Δ	*HMR*-Left PEV eaf3Δ	*HMR*-Left PEV dot1Δ
***p*** ** Value obtained with ON/OFF state frequencies**					
*HMR*-left PEV	0.528388567				
*HMR*-left PEV sas2Δ	0.062231976	0.530081436			
*HMR*-left PEV gcn5Δ	0.301282659	0.019649924	0.530307405		
*HMR*-left PEV eaf3Δ	0.014147096	8.18527E-05	0.065372344	0.527675039	
*HMR*-left PEV dot1Δ	1.52002E-06	0.001121336	1.97997E-07	3.8325E-12	0.534700879
***p*** ** Value obtained with ON to ON and ON to OFF transition frequencies**					
*HMR*-left PEV	0.544136987				
*HMR*-left PEV sas2Δ	0.39855206	0.544292711			
*HMR*-left PEV gcn5Δ	0.00011791	0.000364509	0.577159298		
*HMR*-left PEV eaf3Δ	0.00336358	0.008280341	0.208744276	0.5655856	
*HMR*-left PEV dot1Δ	0.007948409	0.019704415	0.068569191	0.3293684	0.550848699
***p*** ** Value obtained with OFF to ON and OFF to OFF transition frequencies**					
*HMR*-left PEV	0.553451403				
*HMR*-left PEV sas2Δ	0.045994947	0.555828385			
*HMR*-left PEV gcn5Δ	0.014296435	5.58709E-05	0.564304395		
*HMR*-left PEV eaf3Δ	0.024307656	7.89071E-05	0.396543858	0.553306865	
*HMR*-left PEV dot1Δ	0.417230147	0.14783929	0.014243037	0.023580557	0.5705196

Gcn5 is a component of the SAGA histone acetylation enzyme complex, Eaf3 is a component of the NuA4 histone acetylation enzyme complex, and Dot1 is the histone methylation enzyme [27]. To investigate the role of these molecules in the silencing effect, Euchromatin/Euchromatin and *HMR*-left PEV/Euchromatin strains containing deletions of the *GCN5*, *EAF3*, and *DOT1* genes were generated and single cell time-lapse experiments were performed. (Figure 7B−D and Table S6B−D). No epigenetic changes in gene expression were seen in the control strains, which contained *HTB1-EGFP* inserted into the euchromatin region (unpublished data). Although the ratio of ON and OFF cells was comparable between the *gcn5Δ* and wild-type strains, the frequency of ON to OFF transitions was significantly lower in the *gcn5Δ* strain than the wild-type strain. The frequency of OFF to ON transitions was also slightly lower in the mutant strain than in the wild-type strain (Tables 3 and 4). These results coincided with the results of the lineage tree constructed using single cell time-lapse analyses, which showed that the frequency of changes in the expression state from ON to OFF and OFF to ON decreased over multiple generations of the *gcn5Δ* strain (Figure 7B). These data suggested that *GCN5* is involved in regulating the frequency of changes in gene expression over several generations.

The frequencies of the ON to OFF and OFF to ON transitions in the *eaf3Δ* strain were similar to those observed for the *gcn5Δ* strain, but the results of the *sas2Δ* strain did not correlate with those of the *gcn5Δ* and *eaf3Δ* strains (Tables 3 and 4). Deletion of the *DOT1* gene increased the number of cells in the ON state and altered the frequency of the ON to OFF transition slightly; however, the frequency of OFF to ON transition was not affected (Figure 7D, Tables 3 and 4).

The impact of deletion of the *GCN5*, *EAF3*, and *DOT1* genes on gene silencing was then examined using *ADE2* as the reporter instead of EGFP. Wild-type or *gcn5Δ* strains containing the *ADE2* gene at the telomere, *HMR*-left, *HMR*-right, or *HML*-right region were constructed and colony color assays were performed. The wild-type *HMR*-left PEV strain produced pink colonies, whereas the *HMR*-left PEV+*gcn5Δ* strain produced a mixture of colonies containing white, red, sectored, or red-biased colonies (Figure 6). In fact, all of the *gcn5Δ* strains produced the same category of colonies as those produced by the *HMR*-left+*gcn5Δ* strain (Figure 6, Figure S2). These data suggest that disruption of *GCN5* alters the epigenetic control of gene expression at all silencing regions tested. Deletion of the *EAF3* gene in the strain containing *ADE2* at the *HMR*-left region produced colonies that were more red in color than those produced by the *gcn5Δ* strain (Figure 6), which disagreed with the statistical analyses of the single cell experiments. Similar colonies were also observed for the *eaf3Δ* strains in which the marker was inserted at the telomere, *HMR*-right, or *HML*-right region (Figure S2). A weakening of the red color of the colonies was observed following deletion of the *DOT1* gene in all constructs (Figure 6, Figure S2).

Taken together, these results indicate that the acetylation status of histones, which is controlled by histone modification enzymes, exerts an epigenetic influence on gene expression in yeast cells.

### The Spread of Silencing in a Single Cell Is Functionally Correlated Within the *HM* Regions

**Figure 8:**
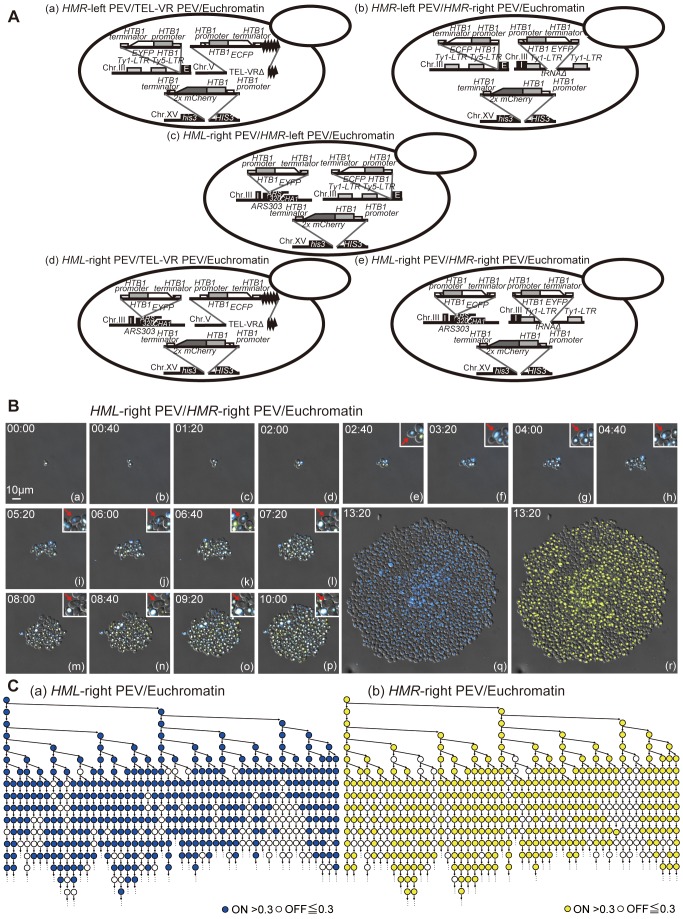
Gene expression states in PEV strains expressing three different fluorescently labeled proteins. (A) Schematic illustration of the yeast strains expressing EYFP, ECFP, and mCherry. (B) Time-lapse images of the *HM*L-right PEV/*HMR*-right PEV/Euchromatin (FUY810) strain. The ECFP signal is shown in blue, the EYFP signal is shown in yellow, and the mCherry signal is shown in red. Overlap of these fluorescent markers is indicated by white coloring. Image stacks were taken at 40 min intervals over a 10 h period and the differential interference contrast image was merged. Panels (q) and (r) show wide focus images that display the ECFP and EYFP signals, respectively. The red arrowhead indicates the same cell in each image. Scale bar, 10 µm. See also Movies S4. (C) Representative lineage trees showing the fluorescence intensities of the ECFP (a) and EYFP (b) signals in the *HML*-right PEV/*HMR*-right PEV/Euchromatin (FUY810) strain tracked in real time. The highest positioned circle on the left side indicates the first single cell; the downward arrowhead pointing from this circle to the right side indicates the daughter cell, and the downward arrow indicates the divided mother cell. Blue and yellow circles indicate a fluorescence intensity >0.3 and white circles indicate a fluorescence intensity <0.3. Not every cell is indicated on this tree due to spatial constraints. Every cell was counted and the data are summarized in Table S7.

To determine whether the spread of silencing within a single cell correlates with the functioning of the different silencing regions, single cell time-lapse experiments were performed using yeast strains expressing three different fluorescent proteins (Figure 8A), namely H2B-EYFP, H2B-ECFP, and H2B-mCherry. All strains contained *HTB1-2x mCherry* at the *HIS3* locus on chromosome XV. The *HMR*-left PEV/TEL-VR PEV/Euchromatin strain contained *HTB1-EYFP* at the *HMR*-left region on chromosome III and *HTB1-ECFP* at the telomere on the right side of chromosome V; the *HMR*-left PEV/*HMR*-right PEV/Euchromatin strain contained *HTB1-ECFP* at the *HMR*-left region of chromosome III and *HTB1-EYFP* at the *tRNA* of the *HMR*-right region of chromosome III; the *HML*-right PEV/*HMR*-left PEV/Euchromatin strain contained *HTB1-EYFP* and *HTB1-ECFP* at the *HML*-right and *HMR*-left regions of chromosome III, respectively; the *HML*-right PEV/TEL-VR PEV/Euchromatin strain contained *HTB1-EYFP* at the *HML*-right region of chromosome III and *HTB1-ECFP* at the telomere on the right side of chromosome V; and the *HML*-right PEV/*HMR*-right PEV/Euchromatin strain contained *HTB1-ECFP* at the *HML*-right region of chromosome III and *HTB1-EYFP* at the *tRNA* of the *HMR*-right region on chromosome III. All strains were examined using single cell time-lapse experiments (Figures 8B, 8C, and S3; Table S7) and correlation analyses were performed (Tables 5 and 6). The correlation between the *HMR*-right and either the *HMR*-left or *HML*-right region was highly significant. A correlation was also observed between the *HML*-right and *HMR*-left regions. However, a correlation between the telomere and either the *HMR*-left or *HML*-right region was not observed. The significance (*p* value) of the probability of two regions behaving independently was larger than 0.1 for comparisons of the TEL-VR PEV and *HM* regions (Table 5). By contrast, the probability was extremely low for comparisons of the *HMR*-left and *HMR*-right regions, the *HMR*-left and *HML*-right regions, and the *HMR*-right and *HML*-right regions (Table 5). These data indicate that the telomere region behaves independently, whereas the *HMR* and *HML* regions behave synchronously with high statistical significance. In Table 6, the left panel displays actual values and the right panel displays the expected appearance frequency under the assumption of no correlation. The actual values exceeded the expected values under no correlation for the ON to ON or OFF to OFF comparisons of the *HMR*-left and *HMR*-right regions, the *HML*-right and *HMR*-left regions, and the *HML*-right and *HMR*-right regions. These data also suggested a positive correlation between the gene expression states of the *HMR*-left and *HMR*-right regions, as well as between the *HMR* and *HML* regions.

**Table 5:** Statistical test for correlation.

	1st col	2nd col	pχ2	psync
(a)	*HMR*-left PEV	TEL-VR PEV	0.255583	0.281914252
(b)	*HMR*-left PEV	*HMR*-right PEV	5.70123 E-10	1.5065E-06
(c)	*HML*-right *PEV*	*HMR*-left PEV	0.000151	0.001747132
(d)	*HML*-right *PEV*	TEL-VR PEV	0.961467	0.518849424
(e)	*HML*-right *PEV*	*HMR*-right PEV	2.2204 E-16	2.01725E-09

**Table 6:** Contingency tables of statistical test for correlation.

(a)	TEL-VR PEV (y)					
*HMR*-left PEV(x)	f (x,y)	ON	OFF	f(x)f(y)/N	ON	OFF
	ON	281	23	ON	277.737	26.2635
	OFF	142	17	OFF	145.26	13.7365

(b)	*HMR*-right PEV(y)					
*HMR-left PEV(x)*	f (x,y)	ON	OFF	f(x)f(y)/N	ON	OFF
	ON	285	33	ON	260.06	57.9379
	OFF	110	55	OFF	134.938	30.0621

(c)	*HMR*-left PEV(y)					
*HML*-right PEV(x)	f (x,y)	ON	OFF	f(x)f(y)/N	ON	OFF
	ON	225	148	ON	209.204	163.796
	OFF	33	54	OFF	48.7957	38.2043

(d)	TEL-VR PEV(y)					
*HML*-right PEV(x)	f(x,y)	ON	OFF	f(x)f(y)/N	ON	OFF
	ON	315	42	ON	314.897	42.1032
	OFF	44	6	OFF	44.1032	5.89681

(e)	*HMR*-right PEV(y)					
*HML*-right PEV(x)	f(x,y)	ON	OFF	f(x)f(y)/N	ON	OFF
	ON	317	44	ON	288.641	72.359
	OFF	46	47	OFF	74.359	18.641

## Discussion

### Functional correlations Between Epigenetic Gene Expression at Different Silencing Regions

In budding yeast, a colony turns red when expression of the *ADE2* gene is repressed. Consistent with a previous report [13], when *ADE2* was inserted close to the telomere, colonies containing red and white sectors were produced [13]. A similar phenotype was also observed when *ADE2* was inserted into the *HMR*-right region, as reported previously [28]. When the reporter gene was changed from *ADE2* to *URA3*, the transformed yeast grew on medium lacking uracil and on medium containing 5-FOA. These data indicate that, despite the presence of the same DNA sequence, different gene expression states occurred simultaneously within the transformed yeast strains, suggesting epigenetic control of gene expression. The results of the previous studies [13],[27] did not show the expression status of an individual cell because mixed populations of cells were used. In addition, the production of a sectored colony indicates that gene expression did not change at every division, but rather that the same expression state was maintained over several generations. If the expression status changed every generation or every few generations, a pink colony would have been produced. In this study, we developed a system to monitor changes in epigenetic gene expression in a single cell across many generations; this technique was used to analyze gene expression at the *HMR*, *HML*, and telomere regions. The results indicated that the gene expression status can change in all of these genetic regions, even after a cell has maintained the same state for several generations, and that the change in expression from the ON to the OFF state is reversible. Although it did not occur for all regions examined in this study, the tendency for genes to switch from the ON to the OFF state was generally more common than the tendency to switch in the opposite direction, which was also demonstrated previously using a pedigree assay [17]. The *HMR*-left region tended to be ON or OFF at random, while the expression status of the *HMR*-right and *HML*-right regions tended to be maintained over many generations. In addition, expression of the telomere tended to be more stable than that of the *HMR*-right or *HML*-right regions.

Pink colonies were produced when the *ADE2* gene was inserted into the *HMR*-left region, while sectored pink colonies were produced when the gene was inserted into the *HMR*-right or *HML*-right regions. A red and white sectored colony was produced when the gene was inserted into the telomere region. These data suggest that different mechanisms underlie the spread of silencing within each region. Two different fluorescent markers were used to determine whether the spread of silencing was consistent in two different regions of a single cell. A perfect match between the expression statuses of the *HMR* and *HML* regions, the *HMR* and telomere regions, or the *HML* and telomere regions could not be found; however, correlations between the expression statuses of the *HMR* and *HML* regions were observed in many cells.

A previous report suggested that the quantity of Sir protein in a single cell might be fixed [29]; therefore, large quantities of Sir protein functioning at one region of the genome may result in a deficit of the protein at other regions. This model would explain the relationship between the rDNA region and the telomere; in other words, it is possible that the silencing level of the rDNA region is inversely correlated with the silencing level of the telomere region [30]. On the other hand, the data presented here demonstrate correlation between the left and right sides of the *HMR*, as well as between the *HMR* and *HML* regions. These data support the results of two previous studies, one of which reported that the E- and I-silencers of *HMR* can form a loop structure [31], and another that showed that the bending of chromosome III causes *HMR* and *HML* to form a large loop structure that eventually brings *HMR* and *HML* close together [32].

### Influence of Histone Modifications on Epigenetic Gene Expression

Formation of the boundary of the telomere silencing region depends on a balance of acetylated and deacetylated histones; disruption of the *SAS2* gene disturbs this balance and allows silencing to spread across a large region of the chromosome [4],[23],[24]. We therefore expected the single cell analyses to show a spread of the silencing region and an increased frequency of cells not expressing the marker gene; however, our result in *HMR*-left region was not perfectly much in this telomere boundary model. Our statistical analysis was not strongly reflected, and the frequencies of the changes in the epigenetic gene expression state from ON to OFF or OFF to ON over multiple generations were increased in lineage of some mother of *sas2Δ*cells (Figure 7A). In future analyses that focus on the age or memory of an individual cell, epigenetic regulation of *SAS2* must be considered.

Disruption of the *GCN5* gene induced a bias of the same gene expression state within a cell. This result was confirmed by an *ADE2* colony assay; deletion of the *GCN5* gene resulted in the production of two different populations of cells (red and white colonies), which also indicates changes in gene expression and suggests that characteristics differed between individual cells, possibly due to changes caused by deletion of *GCN5*. Results of previous studies that used embryonic or induced pluripotent stem cells also suggest that a specific property or characteristic may differ between individual cells [33],[34]. Elucidating the molecular mechanisms that underlie epigenetic modification of gene expression in yeast could contribute to understanding this problem in other organisms.

Eaf3 is important for the formation of the boundary region [7]. In this study, deletion of the *EAF3* gene might not affect the epigenetic status of gene expression and a spread of the silencing region was observed in the *eaf3Δ* strain, as previously reported [7]. Furthermore, single cell analysis showed that the ON state cell increased and altered the frequency of the ON to OFF transition in the *dot1Δ* strain (Tables 3 and 4). Similar results were obtained using the *ADE2* colony assay, which showed that the *dot1Δ* strain increased white colony of phenotype of increasing ON state cell (Figure 6F). In fact, *dot1Δ* cells containing the marker gene in the ON position proliferated according to the anti-silencing mechanism mediated by *DOT1*, as previously reported for a *DOT1* deletion strain [35],[36]. On the other hand, a recent paper reported a positive feedback model in which the H3K79 methylation target of Dot1 was enriched at the ON telomere where it caused disruption of transcriptional silencing [37]. Moreover, another group showed that *dot1Δ* cells establish rapid silencing, and that daughter cells of *dot1Δ* cells established silencing earlier than mother cells [38]. It is difficult to compare directly these results with our results and draw the conclusion that Dot1 function is different at the *HMR* and the telomere [35],[36]; thus, future experiments will be required to determine more precisely the role of Dot1 in epigenetic gene expression.

Further analyses are required to elucidate the molecular mechanisms underlying changes in epigenetic gene expression, including how changes in the acetylation state of histones influence epigenetic control. In addition, the mechanisms controlling gene expression fluctuations at individual silencing regions were different, but similar phenotypes were observed for both the telomere and *HM* regions in an *ADE2* colony assay using strains in which genes encoding histone modifier enzymes were disrupted. This result suggests that changes in the histone modification state have a greater influence on the regulation of gene expression fluctuation than the position of the gene.

### The Importance of Performing Analyses Using a Single Cell

Because conventional large-scale *S. cerevisiae* cultures comprise mixed populations of cells in various states of gene expression, a system for analyzing epigenetic gene expression using single yeast cells was recently introduced [18],[20]. However, previous studies of single yeast cells using this system followed the cells for only a few generations. The technique described here enabled monitoring of single yeast cells for more than 10 generations. Using this system, changes in epigenetic gene expression were shown to be reversible and a histone modification enzyme was shown to control these changes. A functional correlation between different epigenetically regulated regions was also identified. In this study, we also analyzed a phenomenon known as PEV. When the *HTB1-EGFP* gene was inserted into the *HMR*-left, *HMR*-right, *HML*-right, or telomere region, the occurrence of the OFF state of the marker gene expression was much lower than the occurrence of the ON state. Because most conventional epigenetic analyses are performed using a mixed population of cells, it is possible that such experiments are biased towards major phenotypes and potentially miss important minor phenotypes. One of the reasons that the results presented here were not perfectly consistent with those of previous studies may be due to the use of yeast strains of different ages. The abundance of the Sir protein decreases in older cells, while the presence of acetylated histones increases [39].

The results presented here suggest that the epigenetic control of gene expression is not only random but can also be nonrandom. Therefore, new statistical processing methods that enable elucidation of the mechanisms responsible for the epigenetic control of gene expression must be developed.

### The Importance of the Spread of Silencing *in Vivo*

The single cell analysis method described in this study showed that the functioning of the silencing regions differed in individual cells; however, it is unclear why fluctuations in the silencing region are important for controlling epigenetic gene expression. The nature of the genes that control this fluctuation *in vivo* is also unclear. A set of genes with similar and correlated functions form a cluster near the telomere, and expression of these genes may be controlled by the spreading of the silencing effect. Using the single cell analysis system described here, it is possible to identify genes that are controlled in the same cell at the same time. Future experiments will examine how fluctuations in the silencing domain control gene expression at the molecular level, and why fluctuation of the silencing domain in a cell is important.

## Materials and Methods

Full details of the plasmids and primers used in this study will be provided upon request. Standard molecular biology techniques, methods of yeast manipulation, media, and plasmid transformation methods were used.

### Strains and Plasmids

The *S. cerevisiae* strains and the plasmids used in this study are described in Tables S1 and S2.

### Single Cell Imaging and Analysis

Cells were grown in YPD medium at 30°C until the early logarithmic phase. For live imaging, cells were placed in a Y2 microfluidic plate (ONIX). Time-lapse imaging was performed using an Axio Observer Z1 (Carl Zeiss) microscope fitted with a 40× Plan-Neofluar objective lens (NA = 1.3).

### Statistical Analyses of Gene Expression State Transitions

To determine the stability of the nonsilenced (ON) and silenced (OFF) states of individual regions, the frequencies of the following transitions (T) between the ON and OFF states were calculated for individual lineages: T_ON→ON_, T_ON→OFF_, T_OFF→ON_, and T_OFF→OFF_. The stabilities of the ON (q_ON_) and OFF (q_OFF_) states were then quantified as q_ON_ = T_ON→OFF_/(T_ON→ON_+T_ON→OFF_), and q_OFF_ = T_OFF→ON_/(T_OFF→OFF_+T_OFF→ON_). The total frequencies of the transitions from the ON (T_ON_) and OFF (T_OFF_) states were calculated as follows: T_ON_ = T_ON→ON_+T_ON→OFF_, and T_OFF_ = T_OFF→ON_+T_OFF→OFF_. A permutation test was used to determine the statistical significance of differences in the stability of each state between two lineages; the *p* value was calculated as shown in Equation 1.
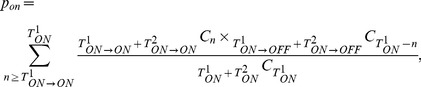
(1)where T^1^_ON→ON_ and T^1^_ON→OFF_ are the transitions for the first lineage, T^2^_ON→ON_ and T^2^_ON→OFF_ are the transitions for the second lineage, and _m_C_n_ is n-combination of m. The same calculations were performed to determine the stability of the OFF state. The data are summarized in Tables 1–6.

### Statistical Tests of Correlation

The gene expression states of two different regions were observed within a single cell simultaneously. To determine the degree of correlation between activity of the individual regions, the frequencies of the ON and OFF states of each region were calculated as f_x_(x) and f_y_(y), and the joint frequency for the two regions was calculated as f_x,y_(x,y); these data are summarized in Tables 5 and 6. Assuming that the null hypothesis of two regions behaving independently is correct, f_x,y_(x,y) is expected to be close to f_x_(x) f_y_(y)/N, where N is the total number of cells observed within one lineage. Deviations from this expectation, which are known to follow a Chi-squared distribution with one degree of freedom, were determined as shown in Equation 2.
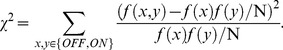
(2)The *p* value associated with χ^2^ (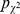) was then calculated using the Chi-squared cumulative distribution. To test the statistical significance of the correlation between behavior of the *HMR*-left and *HMR*-right regions, as well as the *HMR* and *HML* regions, 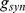 was calculated as f_x,y_(ON,ON)+f_x,y_(OFF,OFF). Assuming that the null hypothesis of independence is correct, the probability of observing (x,y) = (ON, ON) or (x,y) = (OFF, OFF) 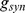 times out of *N* trials should follow the binomial distribution with a success probability (q) equal to [f_x_(ON)f_y_(ON)+f_x_(OFF)f_y_(OFF)]/N^2^. Then, the *p* value (*p_syn_*) was calculated as shown in Equation 3:
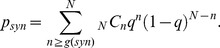
(3)


## Supporting Information

Figure S1 Gene expression states in PEV strains assessed by time-lapse imaging of single yeast cells. (A) Schematic illustration of the yeast strains expressing EGFP. Time-lapse images of the Euchromatin (FUY259) (B), *ty5Δ*:: *NLS-3xEGFP* (FUY260) (C), same as Figure 2.
(TIF)

Figure S2 Position effects at Telomer, *HMR*-right, *HML*-right by *ADE2* colony assay with disruption strain of histone modification enzyme. Position effect of *ADE2* expression results in variegated colonies when inserted at the telomere, *HMR*-left, *HMR*-right, and *HML*-right in the *sir3Δ*, *sas2Δ*, *gcn5Δ*, *eaf3Δ*, and *dot1Δ* strains. A colony color assay was performed using cells carrying telomere-linked *ADE2* ((B) TEL-VR PEV (FUY328), (F) TEL-VR PEV+*sir3Δ* (FUY339), (J) TEL-VR PEV+*sas2Δ* (FUY344), (N) TEL-VR PEV+*gcn5Δ* (FUY789), (R) TEL-VR PEV+*eaf3Δ* (FUY812), (V) TEL-VR PEV+*dot1Δ* (FUY818)), *HMR* right-linked *ADE2* ((C) *HMR*-right PEV (FUY331), (G) *HMR*-right PEV+*sir3Δ* (FUY342), (K) *HMR*-right PEV+*sas2Δ* (FUY347), (O) *HMR*-right PEV+*gcn5Δ* (FUY792), (S) *HMR*-right PEV+*eaf3Δ* (FUY815), (W) *HMR*-right PEV+*dot1Δ* (FUY821)), and *HML* right-linked *ADE2* ((D) *HML*-right PEV (FUY784), (H) *HML*-right PEV+*sir3Δ* (FUY786), (L) *HML*-right PEV+*sas2Δ* (FUY787)), (P) *HML*-left PEV+ *gcn5Δ* (FUY793), (T) *HML*-left PEV+*eaf3Δ* (FUY816), (X) *HML*-left PEV+*dot1Δ* (FUY822)). (A) *ade2* (FUY32), (E) *ade2*+*sir3Δ* (FUY338), (I) *ade2*+*sas2Δ* (FUY343), (M) *ade2*+*gcn5Δ* (FUY788), (Q) *ade2*+*eaf3Δ* (FUY811), and (U) *ade2*+*dot1Δ* (FUY817) were used as controls in this experiment. Freshly grown yeast cells were spread onto SC plates (Adenine 10 µg/ml) and incubated at 30°C for 2–3 d. The white bar indicates 2 mm.
(TIF)

Figure S3 Representative expression lineage trees derived from a single cell. (A) Fluorescence intensity in a cell transformed with *HMR*-left PEV/TEL-VR PEV/Euchromatin (FUY488) and tracked in real-time. The circle on the upper left side indicates the first single cell, the arrowhead pointing from this circle to the right indicates the daughter cell, and the downward arrow indicates the divided mother cell. Yellow (*HMR*-left PEV) and blue (TEL-VR PEV) cells indicate a fluorescence intensity >0.3, and the white cell indicates a fluorescence intensity of <0.3. X (Red) indicates a dead cell. Not every cell is indicated on this tree due to spatial constraints. Every cell was counted and the data are summarized in Table S7A. (B) Fluorescence intensity in a cell transformed with *HMR*-left PEV/*HMR*-right PEV/Euchromatin (FUY492) and tracked in real-time as in (A). Every cell was counted and the data are summarized in Table S7B. (C) Fluorescence intensity in a cell transformed with *HM*L-right PEV/*HMR*-left PEV/Euchromatin (FUY804) and tracked in real-time as in (A). Every cell was counted and the data are summarized in Table S7C. (D) Fluorescence intensity in a cell transformed with *HML*-right PEV/TEL-VR PEV/Euchromatin (FUY806) and tracked in real-time as in (A). Every cell was counted and the data are summarized in Table S7D.
(TIF)

Table S1 Yeast strains used in this study.
(DOCX)

Table S2 Plasmids used in this study.
(XLSX)

Table S3 Fluorescence intensity in PEV strains. (A) Fluorescence intensity in TEL-VR PEV/Euchromatin (FUY355). The circle at on the upper left side indicates the first single cell, which was designated Cell No. 1 in this table (related Figure 5A). (B) Fluorescence intensity in *HMR*-left PEV/Euchromatin (FUY263) (related Figure 5B). (C) Fluorescence intensity in *HMR*-right PEV/Euchromatin (FUY356) (related Figure 5C). (D) Fluorescence intensity in *HML*-right PEV/Euchromatin (FUY795) (related Figure 5D).
(XLSX)

Table S4 Statistical analysis of the reproducibility of transitions of the gene expression state at the *HMR*-left PEV. *HMR*-left PEV (1) : FUY263, *HMR*-left PEV (2) : FUY488, *HMR*-left PEV (3) : FUY492, *HMR*-left PEV (4) : FUY804.
(XLSX)

Table S5 Statistical analysis of the reproducibility of transitions of the gene expression state at the TEL-VR PEV. TEL-VR PEV (1) : FUY355, TEL-VR PEV (2) : FUY488, TEL-VR PEV (3) : FUY806.
(XLSX)

Table S6 Fluorescence intensity in histone-modified enzyme deletion strains. (A) Fluorescence intensity in *HMR*-left PEV/Euchromatin+*sas2Δ* (FUY358) (related Figure 7A). (B) Fluorescence intensity in *HMR*-left PEV/Euchromatin+*gcn5Δ* (FUY797) (related Figure 7B). (C) Fluorescence intensity in *HMR*-left PEV/Euchromatin+*eaf3Δ* (FUY799) (related Figure 7C). (D) Fluorescence intensity in cells transformed with *HMR*-left PEV/Euchromatin+*dot1Δ* (FUY801) (related Figure 7D).
(XLSX)

Table S7 Fluorescence intensity in PEV strains with three different fluorescence proteins. (A) Fluorescence intensity in *HMR*-left PEV/TEL-VR PEV/Euchromatin (FUY488) (related Figure S3A). (B) Fluorescence intensity of *HMR*-left PEV/*HMR*-right PEV/Euchromatin (FUY492) (related Figure S3B). (C) Fluorescence intensity in *HML*-right PEV/*HMR*-left PEV/Euchromatin (FUY804) (related Figure S3C). (D) Fluorescence intensity in cells transformed with *HML*-right PEV/TEL-VR PEV/Euchromatin (FUY806) (related Figure S3D). (E) Fluorescence intensity in *HML*-right PEV/*HMR*-right PEV/Euchromatin (FUY810) (related Figure 8C).
(XLSX)

Movie S1 Time-lapse experiment of Euchromatin/Euchromatin (FUY257) carrying *HTB1-EGFP* inserted at the *TRP1* locus on chromosome IV and *HTB1-2x mCherry* inserted at the *HIS3* locus on chromosome XV (DIC only) (related Figure 2B).
(MOV)

Movie S2 Time-lapse experiment of Euchromatin/Euchromatin (FUY257) carrying *HTB1-EGFP* inserted at the *TRP1* locus on chromosome IV and *HTB1-2x mCherry* inserted at the *HIS3* locus on chromosome XV(Merged DIC and *HTB1-2x mCherry* and *HTB1-EGFP*) (related Figure 2B).
(MOV)

Movie S3 Time-lapse experiment of *HMR*-left PEV/Euchromatin (FUY263) carrying *HTB1-EGFP* inserted at the *HMR*-left region on chromosome III and *HTB1-2x mCherry* inserted at the *HIS3* locus on chromosome XV (Merged DIC and *HTB1-2x mCherry* and *HTB1-EGFP*) (related Figure 3A).
(MOV)

Movie S4 Time-lapse experiment of *HML*-right PEV/*HMR*-right PEV/Euchromatin, which contained *H2B-mcherry*, *H2B-ECFP*, and *H2B-EYFP* in the euchromatin region, the *HML*-right PEV region, and the *HMR*-right PEV region, respectively (Merged DIC and *HTB1-2x mCherry*, *HTB1-ECFP*, and *HTB1-EYFP*) (related Figure 8B).
(MOV)

## Abbreviations

5-FOA: 5-fluoroorotic acid
PEV: Position Effect Variegation
Sir: Silent information regulator
