# Supplementary figures and images for: Single Cell Visualization of Yeast Gene Expression Shows Correlation of Epigenetic Switching between Multiple Heterochromatic Regions through Multiple Generations
Source: PLoS Biol. 2013 Jul 2;11(7):e1001601. doi: 10.1371/journal.pbio.1001601 (PMC3699475; doi:10.1371/journal.pbio.1001601)

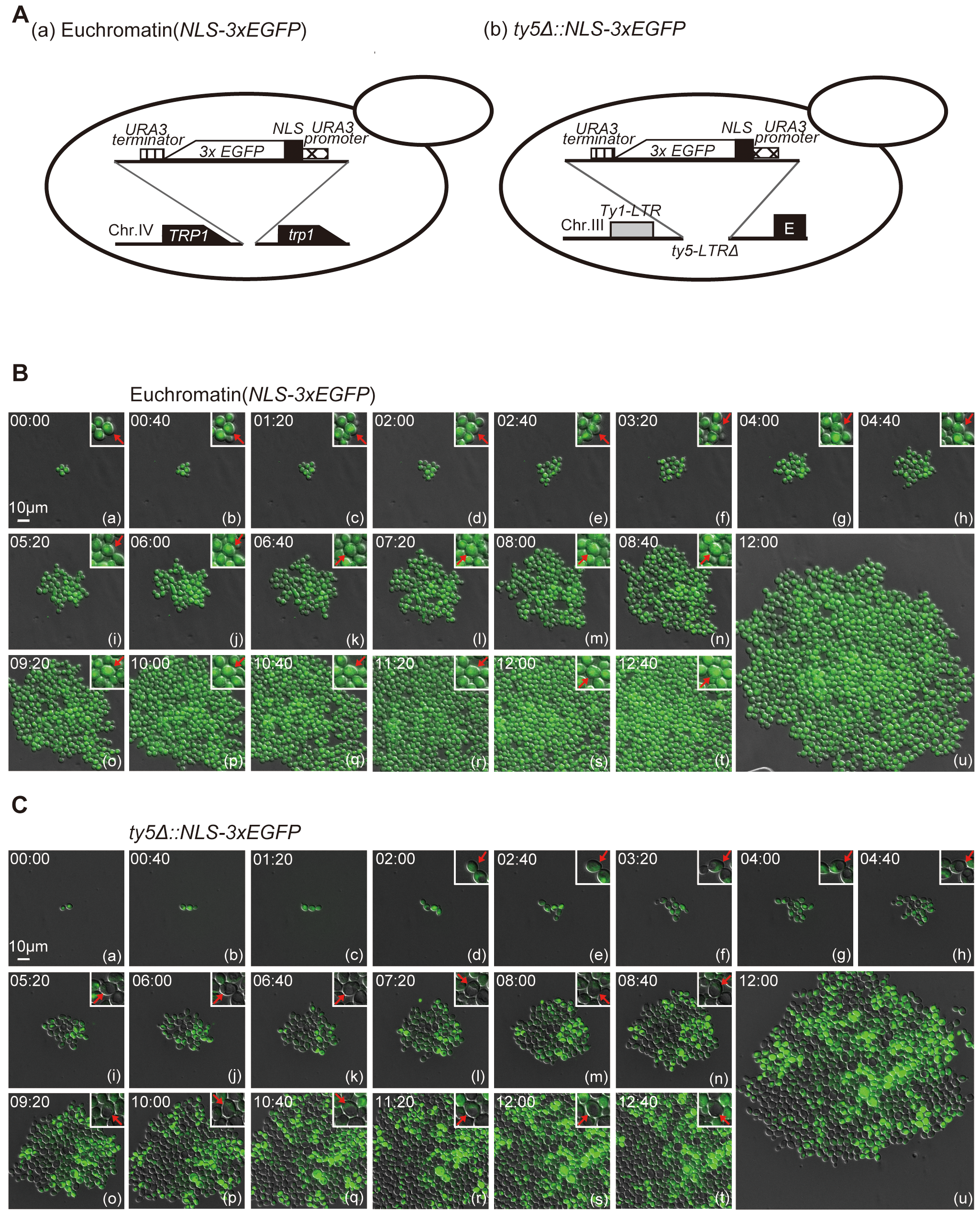

Supplement: Figure S1 — Gene expression states in PEV strains assessed by time-lapse imaging of single yeast cells. (A) Schematic illustration of the yeast strains expressing EGFP. Time-lapse images of the Euchromatin (FUY259) (B), ty5Δ:: NLS-3xEGFP (FUY260) (C), same as Figure 2. (TIF) [file pbio.1001601.s001.tif]

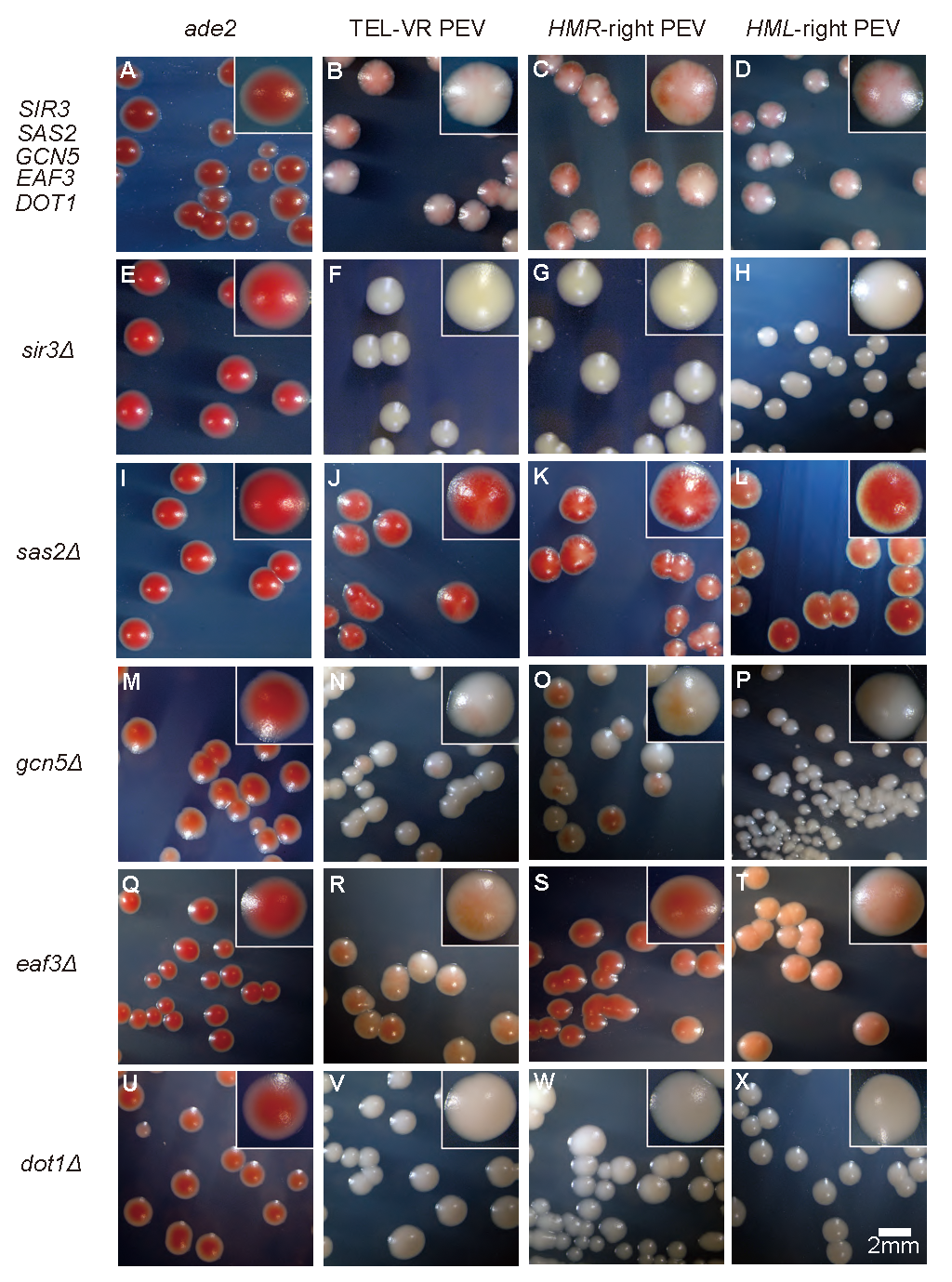

Supplement: Figure S2 — Position effects at Telomer, HMR-right, HML-right by ADE2 colony assay with disruption strain of histone modification enzyme. Position effect of ADE2 expression results in variegated colonies when inserted at the telomere, HMR-left, HMR-right, and HML-right in the sir3Δ, sas2Δ, gcn5Δ, eaf3Δ, and dot1Δ strains. A colony color assay was performed using cells carrying telomere-linked ADE2 ((B) TEL-VR PEV (FUY328), (F) TEL-VR PEV+sir3Δ (FUY339), (J) TEL-VR PEV+sas2Δ (FUY344), (N) TEL-VR PEV+gcn5Δ (FUY789), (R) TEL-VR PEV+eaf3Δ (FUY812), (V) TEL-VR PEV+dot1Δ (FUY818)), HMR right-linked ADE2 ((C) HMR-right PEV (FUY331), (G) HMR-right PEV+sir3Δ (FUY342), (K) HMR-right PEV+sas2Δ (FUY347), (O) HMR-right PEV+gcn5Δ (FUY792), (S) HMR-right PEV+eaf3Δ (FUY815), (W) HMR-right PEV+dot1Δ (FUY821)), and HML right-linked ADE2 ((D) HML-right PEV (FUY784), (H) HML-right PEV+sir3Δ (FUY786), (L) HML-right PEV+sas2Δ (FUY787)), (P) HML-left PEV+ gcn5Δ (FUY793), (T) HML-left PEV+eaf3Δ (FUY816), (X) HML-left PEV+dot1Δ (FUY822)). (A) ade2 (FUY32), (E) ade2+sir3Δ (FUY338), (I) ade2+sas2Δ (FUY343), (M) ade2+gcn5Δ (FUY788), (Q) ade2+eaf3Δ (FUY811), and (U) ade2+dot1Δ (FUY817) were used as controls in this experiment. Freshly grown yeast cells were spread onto SC plates (Adenine 10 µg/ml) and incubated at 30°C for 2–3 d. The white bar indicates 2 mm. (TIF) [file pbio.1001601.s002.tif]

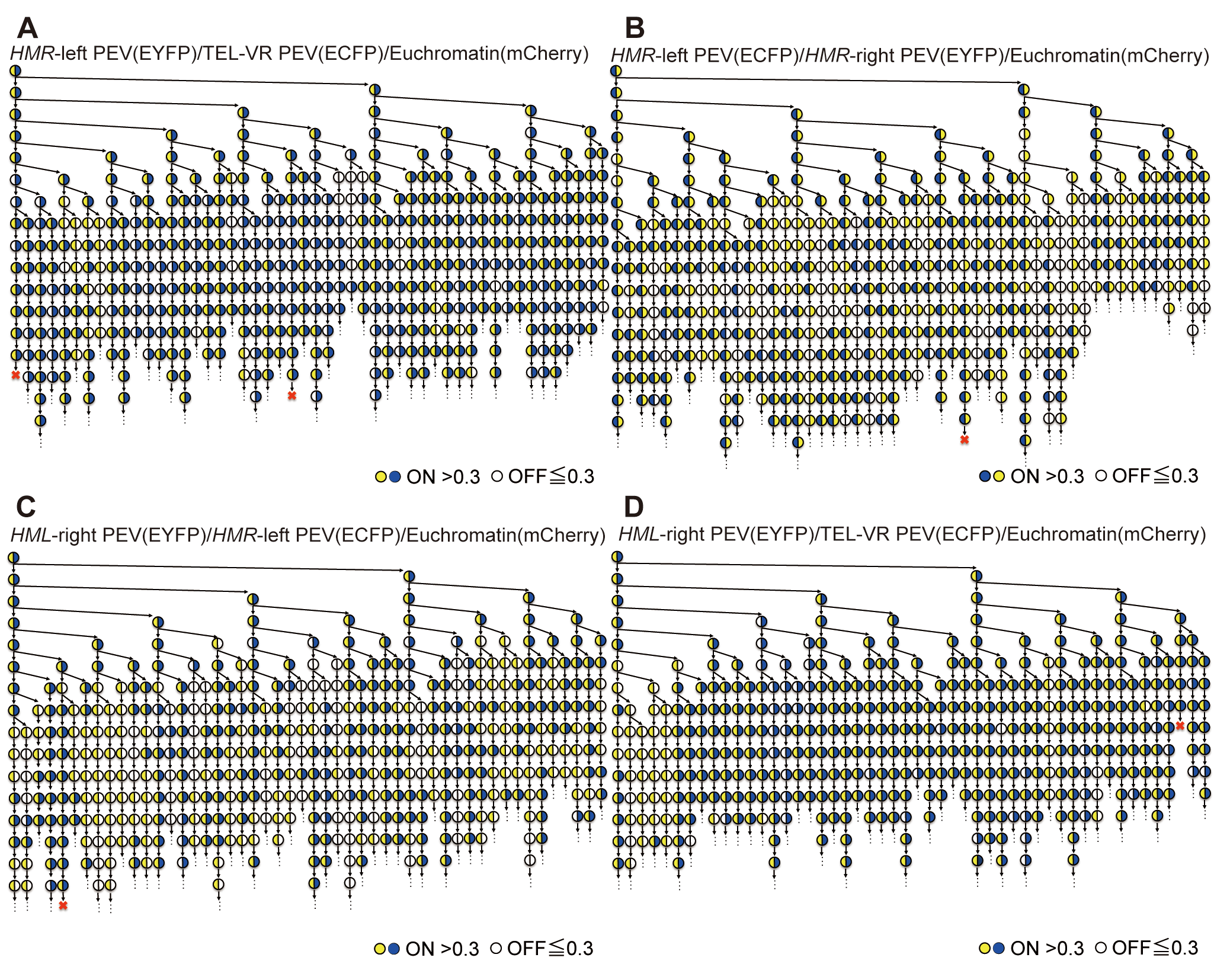

Supplement: Figure S3 — Representative expression lineage trees derived from a single cell. (A) Fluorescence intensity in a cell transformed with HMR-left PEV/TEL-VR PEV/Euchromatin (FUY488) and tracked in real-time. The circle on the upper left side indicates the first single cell, the arrowhead pointing from this circle to the right indicates the daughter cell, and the downward arrow indicates the divided mother cell. Yellow (HMR-left PEV) and blue (TEL-VR PEV) cells indicate a fluorescence intensity >0.3, and the white cell indicates a fluorescence intensity of <0.3. X (Red) indicates a dead cell. Not every cell is indicated on this tree due to spatial constraints. Every cell was counted and the data are summarized in Table S7A. (B) Fluorescence intensity in a cell transformed with HMR-left PEV/HMR-right PEV/Euchromatin (FUY492) and tracked in real-time as in (A). Every cell was counted and the data are summarized in Table S7B. (C) Fluorescence intensity in a cell transformed with HML-right PEV/HMR-left PEV/Euchromatin (FUY804) and tracked in real-time as in (A). Every cell was counted and the data are summarized in Table S7C. (D) Fluorescence intensity in a cell transformed with HML-right PEV/TEL-VR PEV/Euchromatin (FUY806) and tracked in real-time as in (A). Every cell was counted and the data are summarized in Table S7D. (TIF) [file pbio.1001601.s003.tif]
