# Supplementary material for: Single Cell Visualization of Yeast Gene Expression Shows Correlation of Epigenetic Switching between Multiple Heterochromatic Regions through Multiple Generations
Source: PLoS Biol. 2013 Jul 2;11(7):e1001601. doi: 10.1371/journal.pbio.1001601 (PMC3699475; doi:10.1371/journal.pbio.1001601)
Supplement: Table S1 — Yeast strains used in this study. (DOCX) [file pbio.1001601.s004.docx]

Table S1. Yeast strains used in this study.

| Strain |  | Genotype |  | Background | Source |
| --- | --- | --- | --- | --- | --- |
| FUY31 |  | *MATa ADE2 lys2Δ his3-11,15 leu2-3,112 trp1-1 ura3-1* |  | *—* | *W-303 wild type* |
| FUY18 |  | *MATa his4* |  | *—* | *Kamakaka RT* |
| FUY323 |  | *MATa ADE2 lys2Δ his3-11,15 leu2-3,112 trp1-1 ura3-1* VR*::URA3*-TEL |  | FUY31 | *This study* |
| FUY324 |  | *MATa ADE2 lys2Δ his3-11,15 leu2-3,112 trp1-1 ura3-1 ty5Δ::URA3* |  | FUY31 | *This study* |
| FUY316 |  | *MATa ADE2 lys2Δ his3-11,15 leu2-3,112 trp1-1 ura3-1 HMR*-left PEV (*ty5(1)Δ::URA3*) |  | FUY31 | *This study* |
| FUY325 |  | *MATa ADE2 lys2Δ his3-11,15 leu2-3,112 trp1-1 ura3-1 ty5(1)Δ::URA3-*R |  | FUY31 | *This study* |
| FUY326 |  | *MATa ADE2 lys2Δ his3-11,15 leu2-3,112 trp1-1 ura3-1 HMR*-right PEV (*tRNAΔ::URA3*) |  | FUY31 | *This study* |
| FUY327 |  | *MATa ADE2 lys2Δ his3-11,15 leu2-3,112 trp1-1 ura3-1 tRNAΔ::URA3*-R |  | FUY31 | *This study* |
| FUY782 |  | *MATa ADE2 lys2Δ his3-11,15 leu2-3,112 trp1-1 ura3-1 HML*-right PEV (*ARS303-URA3-ARS320*) |  | FUY31 | *This study* |
| FUY783 |  | *MATa ADE2 lys2Δ his3-11,15 leu2-3,112 trp1-1 ura3-1 ARS303-URA3*-R*-ARS320* |  | FUY31 | *This study* |
| FUY32 |  | *MATa ade2-1 LYS2 his3-11,15 leu2-3,112 trp1-1 ura3-1* |  | *—* | *W-303 wild type* |
| FUY328 |  | *MATa ade2-1 LYS2 his3-11,15 leu2-3,112 trp1-1 ura3-1* VR*::ADE2*-TEL |  | FUY32 | *This study* |
| FUY152 |  | *MATa ade2-1 LYS2 his3-11,15 leu2-3,112 trp1-1 ura3-1 ty5Δ::ADE2* |  | FUY32 | *This study* |
| FUY329 |  | *MATa ade2-1 LYS2 his3-11,15 leu2-3,112 trp1-1 ura3-1 HMR*-left PEV (*ty5(1)Δ::ADE2*) |  | FUY32 | *This study* |
| FUY330 |  | *MATa ade2-1 LYS2 his3-11,15 leu2-3,112 trp1-1 ura3-1 ty5(1)Δ::ADE2*-R |  | FUY32 | *This study* |
| FUY331 |  | *MATa ade2-1 LYS2 his3-11,15 leu2-3,112 trp1-1 ura3-1 HMR*-right PEV (*tRNAΔ::ADE2*) |  | FUY32 | *This study* |
| FUY332 |  | *MATa ade2-1 LYS2 his3-11,15 leu2-3,112 trp1-1 ura3-1 tRNAΔ::ADE2*-R |  | FUY32 | *This study* |
| FUY784 |  | *MATa ade2-1 LYS2 his3-11,15 leu2-3,112 trp1-1 ura3-1 HML*-right PEV (*ARS303-ADE2-ARS320*) |  | FUY32 | *This study* |
| FUY785 |  | *MATa ade2-1 LYS2 his3-11,15 leu2-3,112 trp1-1 ura3-1 ARS303-ADE2*-R*-ARS320* |  | FUY32 | *This study* |
| FUY338 |  | *MATa ade2-1 LYS2 his3-11,15 leu2-3,112 trp1-1 ura3-1 sir3Δ::KanMX* |  | FUY32 | *This study* |
| FUY339 |  | *MATa ade2-1 LYS2 his3-11,15 leu2-3,112 trp1-1 ura3-1* VR*::ADE2*-TEL *sir3Δ::KanMX* |  | FUY328 | *This study* |
| FUY341 |  | *MATa ade2-1 LYS2 his3-11,15 leu2-3,112 trp1-1 ura3-1 HMR*-left PEV (*ty5(1)Δ::ADE2*)  *sir3Δ::KanMX* |  | FUY329 | *This study* |
| FUY342 |  | *MATa ade2-1 LYS2 his3-11,15 leu2-3,112 trp1-1 ura3-1 HMR*-right PEV (*tRNAΔ::ADE2*)  *sir3Δ::KanMX* |  | FUY331 | *This study* |
| FUY786 |  | *MATa ade2-1 LYS2 his3-11,15 leu2-3,112 trp1-1 ura3-1 HML*-right PEV (*ARS303-ADE2-ARS320*)  *sir3Δ::KanMX* |  | FUY784 | *This study* |
| FUY343 |  | *MATa ade2-1 LYS2 his3-11,15 leu2-3,112 trp1-1 ura3-1 sas2Δ::KanMX* |  | FUY32 | *This study* |
| FUY344 |  | *MATa ade2-1 LYS2 his3-11,15 leu2-3,112 trp1-1 ura3-1* VR*::ADE2*-TEL *sas2Δ::KanMX* |  | FUY328 | *This study* |
| FUY346 |  | *MATa ade2-1 LYS2 his3-11,15 leu2-3,112 trp1-1 ura3-1 HMR*-left PEV (*ty5(1)Δ::ADE2*)  *sas2Δ::KanMX* |  | FUY329 | *This study* |
| FUY347 |  | *MATa ade2-1 LYS2 his3-11,15 leu2-3,112 trp1-1 ura3-1 HMR*-right PEV (*tRNAΔ::ADE2*)  *sas2Δ::KanMX* |  | FUY331 | *This study* |
| FUY787 |  | *MATa ade2-1 LYS2 his3-11,15 leu2-3,112 trp1-1 ura3-1 HML*-right PEV (*ARS303-ADE2-ARS320*) *sas2Δ::KanMX* |  | FUY784 | *This study* |
| FUY788 |  | *MATa ade2-1 LYS2 his3-11,15 leu2-3,112 trp1-1 ura3-1 gcn5Δ::KanMX* |  | FUY32 | *This study* |
| FUY789 |  | *MATa ade2-1 LYS2 his3-11,15 leu2-3,112 trp1-1 ura3-1* VR*::ADE2*-TEL *gcn5Δ::KanMX* |  | FUY328 | *This study* |
| FUY791 |  | *MATa ade2-1 LYS2 his3-11,15 leu2-3,112 trp1-1 ura3-1 HMR*-left PEV (*ty5(1)Δ::ADE2*)  *gcn5Δ::KanMX* |  | FUY329 | *This study* |
| FUY792 |  | *MATa ade2-1 LYS2 his3-11,15 leu2-3,112 trp1-1 ura3-1 HMR*-right PEV (*tRNAΔ::ADE2*)  *gcn5Δ::KanMX* |  | FUY331 | *This study* |
| FUY793 |  | *MATa ade2-1 LYS2 his3-11,15 leu2-3,112 trp1-1 ura3-1 HML*-right PEV (*ARS303-ADE2-ARS320*) *gcn5Δ::KanMX* |  | FUY784 | *This study* |
| FUY811 |  | *MATa ade2-1 LYS2 his3-11,15 leu2-3,112 trp1-1 ura3-1 eaf3Δ::KanMX* |  | FUY32 | *This study* |
| FUY812 |  | *MATa ade2-1 LYS2 his3-11,15 leu2-3,112 trp1-1 ura3-1* VR*::ADE2*-TEL *eaf3Δ::KanMX* |  | FUY328 | *This study* |
| FUY814 |  | *MATa ade2-1 LYS2 his3-11,15 leu2-3,112 trp1-1 ura3-1 HMR*-left PEV (*ty5(1)Δ::ADE2*)  *eaf3Δ::KanMX* |  | FUY329 | *This study* |
| FUY815 |  | *MATa ade2-1 LYS2 his3-11,15 leu2-3,112 trp1-1 ura3-1 HMR*-right PEV (*tRNAΔ::ADE2*)  *eaf3Δ::KanMX* |  | FUY331 | *This study* |
| FUY816 |  | *MATa ade2-1 LYS2 his3-11,15 leu2-3,112 trp1-1 ura3-1 HML*-right PEV (*ARS303-ADE2-ARS320*) *eaf3Δ::KanMX* |  | FUY784 | *This study* |
| FUY817 |  | *MATa ade2-1 LYS2 his3-11,15 leu2-3,112 trp1-1 ura3-1 dot1Δ::KanMX* |  | FUY32 | *This study* |
| FUY818 |  | *MATa ade2-1 LYS2 his3-11,15 leu2-3,112 trp1-1 ura3-1* VR*::ADE2*-TEL *dot1Δ::KanMX* |  | FUY328 | *This study* |
| FUY820 |  | *MATa ade2-1 LYS2 his3-11,15 leu2-3,112 trp1-1 ura3-1 HMR*-left PEV (*ty5(1)Δ::ADE2*)  *dot1Δ::KanMX* |  | FUY329 | *This study* |
| FUY821 |  | *MATa ade2-1 LYS2 his3-11,15 leu2-3,112 trp1-1 ura3-1 HMR*-right PEV (*tRNAΔ::ADE2*)  *dot1Δ::KanMX* |  | FUY331 | *This study* |
| FUY822 |  | *MATa ade2-1 LYS2 his3-11,15 leu2-3,112 trp1-1 ura3-1 HML*-right PEV (*ARS303-ADE2-ARS320*) *dot1Δ::KanMX* |  | FUY784 | *This study* |
| FUY204 |  | *MATa ADE2 lys2Δ his3-11,15 leu2-3,112 ura3-1* Euchromatin (*trp1-1*:: *HTB1-EGFP-TRP1*) |  | FUY31 | *This study* |
| FUY75 |  | *MATa ADE2 lys2Δ his3-11,15 leu2-3,112 trp1-1 ura3-1 ty1Δ-ty5Δ::URA3* (not PEV) |  | FUY31 | *This study* |
| FUY353 |  | *MATa ADE2 lys2Δ his3-11,15 leu2-3,112 trp1-1 ura3-1* VR*::HTB1-EGFP*-TEL |  | FUY323 | *This study* |
| FUY256 |  | *MATa ADE2 lys2Δ his3-11,15 leu2-3,112 trp1-1 ura3-1 HMR*-left PEV (*ty5(1)Δ::HTB1-EGFP*) |  | FUY75 | *This study* |
| FUY354 |  | *MATa ADE2 lys2Δ his3-11,15 leu2-3,112 trp1-1 ura3-1 HMR*-right PEV (*tRNAΔ::HTB1-EGFP*) |  | FUY327 | *This study* |
| FUY794 |  | *MATa ADE2 lys2Δ his3-11,15 leu2-3,112 trp1-1 ura3-1*  *HML*-right PEV (*ARS303-HTB1-EGFP-ARS320*) |  | FUY783 | *This study* |
| FUY257 |  | *MATa ADE2 lys2Δ leu2-3,112 ura3-1* Euchromatin (*trp1-1*:: *HTB1-EGFP-TRP1*)  Euchromatin (*his3-11,15::HTB1-2x mCherry-HIS3*) |  | FUY204 | *This study* |
| FUY355 |  | *MATa ADE2 lys2Δ leu2-3,112 trp1-1 ura3-1* VR*::HTB1-EGFP*-TEL  Euchromatin (*his3-11,15::HTB1-2x mCherry-HIS3*) |  | FUY353 | *This study* |
| FUY263 |  | *MATa ADE2 lys2Δ leu2-3,112 trp1-1 ura3-1 HMR*-left PEV (*ty5(1)Δ::HTB1-EGFP*)  Euchromatin (*his3-11,15::HTB1-2x mCherry-HIS3*) |  | FUY256 | *This study* |
| FUY356 |  | *MATa ADE2 lys2Δ leu2-3,112 trp1-1 ura3-1 HMR*-right PEV (*tRNAΔ::HTB1-EGFP*)  Euchromatin (*his3-11,15::HTB1-2x mCherry-HIS3*) |  | FUY354 | *This study* |
| FUY795 |  | *MATa ADE2 lys2Δ leu2-3,112 trp1-1 ura3-1 HML*-right PEV (*ARS303-HTB1-EGFP-ARS320*)  Euchromatin (*his3-11,15::HTB1-2x mCherry-HIS3*) |  | FUY794 | *This study* |
| FUY357 |  | *MATa ADE2 lys2Δ leu2-3,112 ura3-1* Euchromatin (*trp1-1*:: *HTB1-EGFP-TRP1*)  Euchromatin (*his3-11,15::HTB1-2x mCherry-HIS3*) *sas2Δ::KanMX* |  | FUY257 | *This study* |
| FUY358 |  | *MATa ADE2 lys2Δ leu2-3,112 trp1-1 ura3-1 HMR*-left PEV (*ty5(1)Δ::HTB1-EGFP*)  Euchromatin (*his3-11,15::HTB1-2x mCherry-HIS3*) *sas2Δ::KanMX* |  | FUY263 | *This study* |
| FUY796 |  | *MATa ADE2 lys2Δ leu2-3,112 ura3-1* Euchromatin (*trp1-1*:: *HTB1-EGFP-TRP1*)  Euchromatin (*his3-11,15::HTB1-2x mCherry-HIS3*) *gcn5Δ::KanMX* |  | FUY257 | *This study* |
| FUY797 |  | *MATa ADE2 lys2Δ leu2-3,112 trp1-1 ura3-1 HMR*-left PEV (*ty5(1)Δ::HTB1-EGFP*)  Euchromatin (*his3-11,15::HTB1-2x mCherry-HIS3*) *gcn5Δ::KanMX* |  | FUY263 | *This study* |
| FUY798 |  | *MATa ADE2 lys2Δ leu2-3,112 ura3-1* Euchromatin (*trp1-1*:: *HTB1-EGFP-TRP1*)  Euchromatin (*his3-11,15::HTB1-2x mCherry-HIS3*) *eaf3Δ::KanMX* |  | FUY257 | *This study* |
| FUY799 |  | *MATa ADE2 lys2Δ leu2-3,112 trp1-1 ura3-1 HMR*-left PEV (*ty5(1)Δ::HTB1-EGFP*)  Euchromatin (*his3-11,15::HTB1-2x mCherry-HIS3*) *eaf3Δ::KanMX* |  | FUY263 | *This study* |
| FUY800 |  | *MATa ADE2 lys2Δ leu2-3,112 ura3-1* Euchromatin (*trp1-1*:: *HTB1-EGFP-TRP1*)  Euchromatin (*his3-11,15::HTB1-2x mCherry-HIS3) dot1Δ::KanMX* |  | FUY257 | *This study* |
| FUY801 |  | *MATa ADE2 lys2Δ leu2-3,112 trp1-1 ura3-1 HMR*-left PEV (*ty5(1)Δ::HTB1-EGFP*)  Euchromatin (*his3-11,15::HTB1-2x mCherry-HIS3*) *dot1Δ::KanMX* |  | FUY263 | *This study* |
| FUY485 |  | *MATa ADE2 lys2Δ his3-11,15 leu2-3,112 trp1-1 ura3-1* VR*::HTB1-ECFP*-TEL |  | FUY323 | *This study* |
| FUY486 |  | *MATa ADE2 lys2Δ leu2-3,112 trp1-1 ura3-1* VR*::HTB1-ECFP*-TEL  Euchromatin (*his3-11,15::HTB1-2x mCherry-HIS3*) |  | FUY485 | *This study* |
| FUY487 |  | *MATa ADE2 lys2Δ leu2-3,112 trp1-1 ura3-1* VR*::HTB1-ECFP*-TEL  Euchromatin (*his3-11,15::HTB1-2x mCherry-HIS3*) *ty1Δ-ty5Δ::URA3*-R (not PEV) |  | FUY486 | *This study* |
| FUY488 |  | *MATa ADE2 lys2Δ leu2-3,112 trp1-1 ura3-1* VR*::HTB1-ECFP*-TEL  Euchromatin (*his3-11,15::HTB1-2x mCherry-HIS3*) *HMR*-left PEV (*ty5(1)Δ::HTB1-EYFP*) |  | FUY487 | *This study* |
| FUY489 |  | *MATa ADE2 lys2Δ his3-11,15 leu2-3,112 trp1-1 ura3-1 HMR*-left PEV (*ty5(1)Δ::HTB1-ECFP*) |  | FUY75 | *This study* |
| FUY490 |  | *MATa ADE2 lys2Δ his3-11,15 leu2-3,112 trp1-1 ura3-1*  *HMR*-left PEV (*ty5(1)Δ::HTB1-ECFP*) *tRNAΔ::URA3*-R |  | FUY489 | *This study* |
| FUY491 |  | *MATa ADE2 lys2Δ his3-11,15 leu2-3,112 trp1-1 ura3-1*  *HMR*-left PEV (*ty5(1)Δ::HTB1-ECFP*) *HMR*-right PEV (*tRNAΔ::HTB1-EYFP*) |  | FUY490 | *This study* |
| FUY492 |  | *MATa ADE2 lys2Δ leu2-3,112 trp1-1 ura3-1 HMR*-left PEV (*ty5(1)Δ::HTB1-ECFP*)  *HMR*-right PEV (*tRNAΔ::HTB1-EYFP*) Euchromatin (*his3-11,15::HTB1-2x mCherry-HIS3*) |  | FUY491 | *This study* |
| FUY802 |  | *MATa ADE2 lys2Δ leu2-3,112 trp1-1 ura3-1 HMR*-left PEV (*ty5(1)Δ::HTB1-ECFP*)  Euchromatin (*his3-11,15::HTB1-2x mCherry-HIS3*) |  | FUY489 | *This study* |
| FUY803 |  | *MATa ADE2 lys2Δ leu2-3,112 trp1-1 ura3-1 HMR*-left PEV (*ty5(1)Δ::HTB1-ECFP*)  Euchromatin (*his3-11,15::HTB1-2x mCherry-HIS3*) *ARS303-URA3*-R*-ARS320* |  | FUY802 | *This study* |
| FUY804 |  | *MATa ADE2 lys2Δ leu2-3,112 trp1-1 ura3-1 HMR*-left PEV (*ty5(1)Δ::HTB1-ECFP*)  Euchromatin (*his3-11,15::HTB1-2x mCherry-HIS3*) *HML*-right PEV (*ARS303-HTB1-EYFP-ARS320*) |  | FUY803 | *This study* |
| FUY805 |  | *MATa ADE2 lys2Δ leu2-3,112 trp1-1 ura3-1* VR*::HTB1-ECFP*-TEL  Euchromatin (*his3-11,15::HTB1-2x mCherry-HIS3*) *ARS303-URA3*-R*-ARS320* |  | FUY486 | *This study* |
| FUY806 |  | *MATa ADE2 lys2Δ leu2-3,112 trp1-1 ura3-1* VR*::HTB1-ECFP*-TEL  Euchromatin (*his3-11,15::HTB1-2x mCherry-HIS3*) *HML*-right PEV (*ARS303-HTB1-EYFP-ARS320*) |  | FUY805 | *This study* |
| FUY807 |  | *MATa ADE2 lys2Δ his3-11,15 leu2-3,112 trp1-1 ura3-1*  *HML*-right PEV (*ARS303-HTB1-ECFP-ARS320*) |  | FUY783 | *This study* |
| FUY808 |  | *MATa ADE2 lys2Δ leu2-3,112 trp1-1 ura3-1 HML*-right PEV (*ARS303-HTB1-ECFP-ARS320*)  Euchromatin (*his3-11,15::HTB1-2x mCherry-HIS3*) |  | FUY807 | *This study* |
| FUY809 |  | *MATa ADE2 lys2Δ leu2-3,112 trp1-1 ura3-1 HML*-right PEV (*ARS303-HTB1-ECFP-ARS320*)  Euchromatin (*his3-11,15::HTB1-2x mCherry-HIS3*) *tRNAΔ::URA3*-R |  | FUY808 | *This study* |
| FUY810 |  | *MATa ADE2 lys2Δ leu2-3,112 trp1-1 ura3-1 HML*-right PEV (*ARS303-HTB1-ECFP-ARS320*)  Euchromatin (*his3-11,15::HTB1-2x mCherry-HIS3*) *HMR*-right PEV (*tRNAΔ::HTB1-EYFP*) |  | FUY809 | *This study* |
| FUY259 |  | *MATa ADE2 lys2Δ his3-11,15 leu2-3,112 ura3-1* Euchromatin (*trp1-1::NLS-3x EGFP-TRP1*) |  | FUY31 | *This study* |
| FUY260 |  | *MATa ADE2 lys2Δ his3-11,15 leu2-3,112 trp1-1 ura3-1 ty5Δ::NLS-3x EGFP* |  | FUY75 | *This study* |
